# Fragment library screening by X-ray crystallography and binding site analysis on thioredoxin glutathione reductase of *Schistosoma mansoni*

**DOI:** 10.1038/s41598-024-52018-2

**Published:** 2024-01-18

**Authors:** Lauro Ribeiro de Souza Neto, Bogar Omar Montoya, José Brandão-Neto, Anil Verma, Sebastian Bowyer, José Teófilo Moreira-Filho, Rafael Ferreira Dantas, Bruno Junior Neves, Carolina Horta Andrade, Frank von Delft, Raymond J. Owens, Nicholas Furnham, Floriano Paes Silva-Jr

**Affiliations:** 1 LaBECFar - Laboratory of Experimental and Computational Biochemistry of Drugs, Oswaldo Cruz Institute, FIOCRUZ, Rio de Janeiro, Brazil; 2 Diamond Light Source Ltd, Harwell Science and Innovation Campus, Harwell, UK; 3 Research Complex at Harwell, Harwell Science and Innovation Campus, Harwell, UK; 4 Division of Structural Biology, The Wellcome Centre for Human Genetics, University of Oxford, Oxford, UK; 5 Department of Infection Biology, London School of Hygiene and Tropical Medicine, London, UK; 6 LabMol - Laboratory for Molecular Modeling and Design, Faculty of Pharmacy, Universidade Federal de Goiás, Goiânia, Brazil; 7 Laboratory of Cheminformatics, Faculty of Pharmacy, Universidade Federal de Goiás, Goiânia, Brazil; 8 CRAFT - Center for Research and Advancement of Fragments and Molecular Targets, University of São Paulo, São Paulo, Brazil; 9 Centre for Medicines Discovery, University of Oxford, Oxford, UK; 10 Department of Biochemistry, University of Johannesburg, Johannesburg, South Africa; 11 Structural Biology, Rosalind Franklin Institute, Harwell, UK

**Keywords:** Biophysics, Biotechnology, Chemical biology, Computational biology and bioinformatics, Drug discovery, Structural biology, Parasitic infection, Biochemistry, Enzymes, Structural biology

## Abstract

Schistosomiasis is caused by parasites of the genus *Schistosoma*, which infect more than 200 million people. Praziquantel (PZQ) has been the main drug for controlling schistosomiasis for over four decades, but despite that it is ineffective against juvenile worms and size and taste issues with its pharmaceutical forms impose challenges for treating school-aged children. It is also important to note that PZQ resistant strains can be generated in laboratory conditions and observed in the field, hence its extensive use in mass drug administration programs raises concerns about resistance, highlighting the need to search for new schistosomicidal drugs. Schistosomes survival relies on the redox enzyme thioredoxin glutathione reductase (TGR), a validated target for the development of new anti-schistosomal drugs. Here we report a high-throughput fragment screening campaign of 768 compounds against *S. mansoni* TGR (*Sm*TGR) using X-ray crystallography. We observed 49 binding events involving 35 distinct molecular fragments which were found to be distributed across 16 binding sites. Most sites are described for the first time within *Sm*TGR, a noteworthy exception being the “doorstop pocket” near the NADPH binding site. We have compared results from hotspots and pocket druggability analysis of SmTGR with the experimental binding sites found in this work, with our results indicating only limited coincidence between experimental and computational results. Finally, we discuss that binding sites at the doorstop/NADPH binding site and in the SmTGR dimer interface, should be prioritized for developing SmTGR inhibitors as new antischistosomal drugs.

## Introduction

Schistosomiasis is a parasitic disease caused by trematode worms of the genus *Schistosoma*^1^. It affects more than 200 million people in 78 countries, and it is estimated that 700 million people are living at risk of infection^2,3^. Schistosomiasis causes around 200,000 deaths annually, although the years of life lost due to the morbidity caused by chronic infections are considered the major problem of schistosomiasis^4–6^. Preventive chemotherapy with praziquantel (PZQ) in endemic areas via mass drug administration (MDA) programs is schistosomiasis main control measure recommended by World Health Organization (WHO)^3,7,8^. Although PZQ is a safe and effective drug against all forms of schistosomiasis^9^, its extensive use in MDA programs raises concerns about resistance^10^. Indeed, schistosomes can develop PZQ resistance under certain laboratory conditions and there are field reports suggesting a potential reduced efficacy of PZQ in endemic areas^11–16^. However, the latter may be explained by many factors other than resistant parasites, such as low MDA coverage, inadequate dosing, rapid re-infection, and high worm burden^17–20^. However, PZQ is also ineffective against juvenile worms and size and taste issues with its pharmaceutical forms impose challenges for treating school-aged children^21^. Hence, there is an urgent need for discovering new antischistosomal drugs.

Living in the venous system of their definitive hosts, schistosomes are exposed to reactive oxygen species (ROS) produced during the digestion of red blood cells or by cells of the immune system of the hosts. Therefore, schistosomes require mechanisms to maintain their redox homeostasis^22^. Most eukaryotes have two systems for ROS neutralization. In these systems, nicotinamide adenine dinucleotide phosphate (NADPH) acts as a source of reducing equivalents through two flavoenzymes oxidoreductases: glutathione (GSH) reductase (GR, EC 1.8.1.7) and thioredoxin (Trx) reductase (TR, EC 1.8.1.9)^23^. However, in contrast to their definitive hosts, schistosomes completely depend on a single multifunctional enzyme, thioredoxin glutathione reductase (TGR), to maintain reduced forms of both GSH and Trx^24–26^ since they lack catalase and show very low levels of glutathione peroxidase^27^. TGR is a validated and promising drug target, as suppression of its expression causes the death of the parasite^23^.

The *S. mansoni* TGR (*Sm*TGR) (EC 1.8.1.9) is a homodimeric flavoprotein with a head-to-tail orientation between monomers. The flavin adenine dinucleotide (FAD) cofactor is found within a subdomain formed by Tyr108–Thr257 and Val391–Thr461 while NADPH binds to an adjacent site (Gly258–Val358 and Lys364–Ala390)^28^. Like all mammalian TR, *Sm*TGR is a selenoprotein carrying the amino acid selenocysteine (Sec597). Paired with Cys596, this residue directly participates in enzymatic catalysis together with FAD and at least two other cystine pairs (Cys28-S–S-Cys31 and Cys154-S–S-Cys159) with redox activity^29^.

A complex catalysis mechanism, dependent on the presence of NADPH, has been proposed involving three active sites distributed in the TrxR and the Grx domains of the enzyme^30,31^. Knowing this mechanism opened a window of opportunity for the design of strategies aimed at the discovery and development of new drugs.

Fragment-based drug discovery (FBDD) has been established as an efficient strategy for identifying starting points in a drug discovery campaign^32–37^. It involves the screening of small and less complex compounds, generally with molecular weight (MW) < 300 Da and < 20 heavy atoms that can then be used as starting points, combined with knowledge of how they bind to the target, to generate larger more potent inhibitors^38,39^. While the success of fragment-based approaches is unquestionable, they are still largely unexplored for neglected tropical diseases (NTDs)^40^.

In this work, we describe the unprecedented screening of a poised library of 768 molecular fragments against *Sm*TGR using X-ray crystallography. Several new fragment-sized ligands were identified binding to 16 distinct sites across the enzyme. We also performed hotspot and druggability analysis on the high-resolution SmTGR structure determined by us and compared this with the experimental fragment binding sites found by our FBDD campaign. Finally, we discuss the most promising druggable sites for focusing on new SmTGR inhibitors design.

## Materials and methods

### Experimental methods

#### RNA extraction and cDNA synthesis

Swiss Webster mice (~ 60 days) were inoculated with 400 cercariae of *S. mansoni* (BH strain), provided by the Laboratory of Malacology (IOC/Fiocruz), by subcutaneous injection in the dorsal region. After 42 days of infection, the animals were euthanized, and the adult worms (male and female) obtained by perfusion (0.9% NaCl, 10 U/mL of heparin) of the mesenteric and hepatic portal veins^41^. These steps were carried out in accordance with current national legislation for accessing genetic resources (SisGen registration ACC9A7C) and all experimental protocols were approved by the Commission for Ethics in the Use of Animals (CEUA /IOC/ FIOCRUZ, Brazil; License number L-039/2018-A2). Animals were euthanized by carbon dioxide inhalation. All methods are reported in accordance with ARRIVE guidelines (https://arriveguidelines.org). Then, the worms were transferred to a Petri dish containing DMEM medium supplemented with 10% fetal bovine serum and kept in an incubator (37 °C, 5% CO_2_) overnight. Total RNA was extracted from homogenized adult worms using TRIzol reagent (Life Technologies, USA) according to manufacturer instructions. cDNA was synthesized from total RNA using SuperScript™ III First-Strand Synthesis System (Life Technologies).

#### Cloning and expression

Specific primers for amplification of truncated form of *Sm*TGR (*Sm*TGR ^U597_G598del^) from cDNA were designed for cloning into pOPINS3C^42^ cut with KpnI and HindII using InFusion cloning method from Clontech (Clontech, USA). The sequences of the PCR primers are: *Sm*TGRfwd 5’ aagttctgtttcagggcccgCCTCCAGCTGATGGAACATC 3’ and *Sm*TGRrev 5’ atggtctagaaagctttaGCAACCGCTCACTATGGGC 3’. Although ordering a synthetic gene is a standard approach, we capitalized here on further ongoing work on schistosomal proteins and therefore built an *S. mansoni* cDNA library. Moreover, in our work we felt no need for a codon-optimized expression construct for *Sm*TGR, since the truncated cDNA sequence expressed well in the insect cells, obviating the need to purchase it. This truncated protein lacks the last two amino acids (U597 and G598) at C-terminal. Although it is a truncated version, this enzyme still has catalytic activity and has little change in the potency of some inhibitors such as auranofin, potassium antimony tartrate (PAT) and Furoxan^23^. The same occurs when comparing the Km values for natural (ex: GSSG, Trx, H_2_O_2_) and synthetic (Ex: DTNB) substrates between the wild and Sec597Cys forms^31^. The pOPINS3C vector introduces an N-terminal histidine tag along with a SUMO fusion protein that enhances protein expression and a S3C cleavage site connecting the tag to the *Sm*TGR (N-terminal-6xHis-SUMO-S3C-*Sm*TGR).

For the sake of simplicity, from this point on *Sm*TGR^U597_G598del^ will be referred to as *Sm*TGR only. The resulting *Sm*TGR expression construct was sequenced for correct insertion of the target sequence and was then used for construction of baculovirus by co-transfection of insect *Sf*9 cells with 500 ng of construct DNA and 250 ng of bacmid^43,44^ in a volume of 2.5 μL, and 50 μL of *Sf*900II medium. After gently mixing, 1.5 μL of FuGene HD (Promega Corporation, USA) transfection reagent was pipetted directly into the solution, which was incubated for 30 min at room temperature. After this period, the transfection mix was added to 5 × 10^5^ *Sf*9 cells in 0.5 mL of *Sf*900II and gently rocked to allow homogeneous spread. The transfected cells were then incubated for 7 days at 27 °C to generate the P0 viral stock. As a control of the transfection process, a construct containing green fluorescent protein (GFP) was also used following the same procedure.

##### Small-scale expression

After the 7-day period, a new set of *Sf*9 cells (10^6^ cells in 0.5 mL of medium *Sf*900II) was inoculated with 50 μL of P0 viral stock, storing the rest of this stock at 4 °C. The generation of P1 viral stock followed the same method: a new set of insect cells (10^6^ cells in 3 mL of medium) was infected with 30 μL from P0. After 3 days shaking at 28 °C, culture medium was harvested by centrifugation at 6000 × *g* for 15 min and a small sample of the supernatant was analyzed by Western blot with anti-His antibody (Sigma-Aldrich, USA) to check for intracellular expression of 6xHis-SUMO-S3C-*Sm*TGR.

##### Large-scale expression

For scaling-up to a larger volume of expression, a fresh P2 inoculum had to be prepared first. For this purpose, 25 mL of medium containing 10^6^ cells/mL were inoculated with 200 μL P1 viral stock. After 7 days shaking at 27 °C, the culture was centrifuged at 1000 × *g* for 10 min. The last step to obtain P2, the supernatant was filtered with 0.22 μm membrane and stored in a dark container at 4 °C. For the expression procedure, 2.5 L of medium containing 10^6^ cells/mL were infected with 2.5 mL of P2. After shaking at 200 rpm for 3 days at 27 °C, cells were harvested at 6000 × *g* for 15 min and kept at -80 °C.

##### SmTGR purification

Cellular extract from large-scale expression in *Sf*9 insect cells was used as the starting material to purify 6xHis-SUMO-S3C-*Sm*TGR. Harvested cells were resuspended in lysis buffer (50 mM Tris pH 7.5, 500 mM NaCl, 30 mM imidazole, 0.2% Tween) supplemented with 100 U/mL Benzonase and 50 µL per gram of pellet of a protease inhibitor cocktail (P8849, Sigma-Aldrich, USA). All subsequent steps were carried out at 4 °C unless stated otherwise. Cell lysis was performed using a sonicator in short pulses of 10 s with pause intervals of 15 s, for a duration of 10 min. Cellular debris was removed by centrifugation at 30 000 × g for 30 min. The supernatant was loaded into a 5 mL HisTrap FF column (Cytiva, UK) equilibrated with wash buffer (50 mM Tris pH 7.5, 500 mM NaCl, 30 mM imidazole). This buffer was used to wash the chromatographic column prior to elution of 6xHis-SUMO-S3C-*Sm*TGR in elution buffer (50 mM Tris pH 7.5, 500 mM NaCl, 500 mM imidazole). The SUMO tag was cleaved from the 6xHis-SUMO-S3C-*Sm*TGR using S3C protease^45^. The mixture was then purified by reverse Ni-affinity chromatography to obtain *Sm*TGR protein. The protein was concentrated and injected onto a HiLoad 16/60 Superdex 200 pg column (Cytiva, UK) and eluted with Gel filtration buffer (Tris 20 mM pH7.5, NaCl 200 mM). Protein containing fractions were analyzed by 4–10% SDS-PAGE (NuPage, Invitrogen) for purity and quantity. Protein purity was estimated using ImageJ software^46^. Fractions containing the protein of interest were pooled and concentrated for subsequent experiments. Protein quantification was made using UV absorbance and theoretical molar extinction coefficients. The protein batch was kept frozen in -80 °C until the crystallization trials.

##### SmTGR crystallization

An initial screening of ideal conditions for crystallization was performed in microscale. For this purpose, purified *Sm*TGR was concentrated to 12 mg/mL using a 30 kDa of molecular weight cutoff Vivaspin Concentrator (Sigma-Aldrich, USA). Crystallization trials were set up at 20 °C, aided by the Mosquito Xtal3 (SPT Labtech, UK) liquid handling platform. The sitting drops used to grow crystals were set up in 96-well SwissCi MRC 3 lens crystallization plates (SwissCi, UK) and screened against BCS, JCSG-plus, Morpheus, SG1 and PGA screen kits (Molecular Dimensions, UK). The drops contained 100 nL of protein and 100 nL precipitant solution equilibrated against 30 µL reservoirs by vapor diffusion. Among 75 crystallizable conditions, we selected condition C12 from BCS screen kit, based on the size, shape and diffraction resolution of the preliminarily tested crystals. The C12 solution contained 0.1 M calcium chloride dihydrate, 0.1 M magnesium chloride hexahydrate, 0.1 M PIPES pH 7.0 and 22.5% v/v PEG Smear Medium (5.625% w/v PEG 3350, 5.625% w/v PEG 4000, 5.625% w/v PEG 2000, and 5.625% w/v PEG 5000 MME).

Crystallization trials were repeated using C12 solution to accumulate approximately 800 individual crystals, with each of the SwissCi three well lenses being used to setup with 100 nL of 12 mg/mL of purified protein solution, 100 nL of C12 solution, and 100 nL of previously prepared protein seeds. The seeds were prepared from crystals produced during the crystallization trials using the Seed Bead kit (Hampton research, USA). Crystal growths were monitored in a Rockimager 1000 imaging system (Formulatrix, USA), using transmitted light, UV, and cross polarization cameras. The crystals grew during 72 h at 20 °C, and the images were acquired once every 12 h.

#### Soaking and crystal mounting

The 768 compounds from Diamond-SGC Poised Library (DSPL) were stored in a 500 mM stock solution diluted in 100% DMSO^47^. The screening was performed by the crystal soaking method using the Echo 550 acoustic liquid handling (Labcyte, Inc., USA) to dispense 75 nL of compounds directly to drops in the crystallization plates with 300 nL of final volume. The crystal mounting process was done with the aid of a Shifter microscope stage^48^ mounted over a stereoscopic microscope. The crystals were harvested using polymeric loops (MiTeGen, USA) and subsequently frozen in liquid nitrogen. The loops were stored in liquid nitrogen until the data collection.

#### *X-ray *crystallography* data collection and processing*

Data were collected at the beamline I04-1 at 100 K and processed with the fully automated pipelines at Diamond Light Source^49^, which variously combine XDS^50^, xia2^51^, autoPROC^52^ and DIALS^53^ and select resolution limits algorithmically. Manual curation of processing parameters was not applied. Further analysis was performed through XChemExplorer^54^. For each dataset, the version of processed data was selected by the default XChemExplorer score and electron density maps were generated with Dimple^55^ using our previously determined high-resolution structure (PDB ID: 8PDD) as the ground-state reference model. Data analysis revealed three highly related crystals forms: one in C121 space group consisting of one *Sm*TGR protomer in the asymmetric unit and another two both in P121 (one form consisting of the *Sm*TGR constitutive homodimer in the asymmetric unit; the other where the axis of the asymmetric unit had shifted such that the asymmetric unit contained one *Sm*TGR protomer from one homodimer unit and one *Sm*TGR protomer from a second homodimer unit related by crystallographic symmetry). At the time of processing, XChemExplorer, and PanDDA^56^ in particular, assumed crystal form uniformity, therefore the crystal datasets were manually checked and grouped by crystal form. For each grouped dataset ligand-binding events were identified using PanDDA, and ligands were modelled into PanDDA-calculated event maps using Coot^57^. Restraints were calculated with GRADE v. 1.2.19 (Global Phasing Ltd., Cambridge, United Kingdom, 2010), structures were refined with Refmac^58^, and models and quality annotations manually reviewed. For all data sets where a fragment hit was identified, models were inspected and corrected using a combination of Coot and Phenix Refine^59^ to make them deposition ready. Coordinates, structure factors and fully refined and modelled structures for all data sets where a fragment hit was identified are deposited in the Protein Data Bank.

#### *SmTGR *inhibition* assays*

The reducing activity of *Sm*TGR on 5,5'-dithio-bis-[2-nitrobenzoic acid] (DTNB) was carried out according to a method previously described^60^. The reaction medium contained potassium phosphate buffer, 100 mM, pH 7.4; 0.5 mM NADPH and 0.5 mM DTNB. The assay was performed using 384-well microplates with a final volume of 50 µl at 37 °C. The amount of protein (*Sm*TGR) used in the assay was 0.05 µg/µL. To test the inhibition potential of the compounds on the enzyme, *Sm*TGR was incubated for 10 min with the test compounds at 100 µM, auranofin (positive control) at 100 µM or 1% DMSO (negative control). The incubation took place in the presence of all reagents except NADPH, which was used to trigger the reaction. The appearance of the product was continuously recorded by the FlexStation 3 multimode microplate reader (Molecular Devices, USA). Activity was measured following the appearance of thionitrobenzoic acid (TNB), which has an absorbance peak at 412 nm and a molar extinction coefficient of 13.6 mM^−1^.cm^−1^^61^. The tests were also carried out in the absence of the enzyme to discount the contribution of the spontaneous reaction of the substrates to the rate of appearance of TNB^23^. The results were expressed as percentage activity inhibition considering the mean activity of the control group as 0%. The assays were performed in duplicate or in triplicate if the compound inhibited more than 35% enzyme activity.

### Computational methods

#### Analysis of fragment screening results

The dataset was prepared and curated using Schrödinger Maestro Protein Preparation Wizard tool (Schrödinger Release 2022–2) using the following steps: (i) hydrogen atoms were added (pH = 7.4 ± 0.5) and (ii) the protonation and tautomeric states of the residues were defined at pH 7.4 (Schrödinger, LLC, New York, NY, 2014). All images of *Sm*TGR structure throughout this work were generated with Maestro Schrödinger (Release 2022–2) unless otherwise stated.

A rigid body superposition of *Sm*TGR-ligands complexes was performed using UCSF Chimera^62^. This superposition was used to compare the relative positions of *Sm*TGR binding sites with structural features and other ligand positions. Therefore, the aligned structures were used to map the binding sites where multiple (n ≥ 1) fragments were observed (*i.e.*, cluster). The term cluster was adopted to refer to sets of fragments bound to the same binding site. Two fragments were said to be bound in the same site when at least one of their atoms was observed in a range of 5 Å from each other. Residues were included as part of a binding site if they were within 5 Å from the center of mass of any atom of the observed bound fragments.

#### Analysis of hotspots

The FTmap online tool (https://ftmap.bu.edu/) was used to characterize binding hotspots^63^. It uses a set of 16 small organic molecules, many of them organic solvents, to probe enzyme surface^63^. These regions are assumed to be prominent binding sites, rich in structural features with a major role in molecular recognition^64–66^. The hotspot analysis steps include a (1) rigid-body fragment docking, (2) minimization and rescoring, (3) clustering and ranking, (4) determination the consensus site and (5) characterization of the binding site^64^. FTMap simulations were conducted on a dimeric crystal structure of *Sm*TGR. The reference model (PDB: 8PDD) used in this analysis consisted in an apoenzyme structure of *Sm*TGR with FAD cofactor in both subunits. The model under PDB ID 2X99 was used to extract NADPH coordinates.

#### Pocket prediction and druggability score

Following the hotspot prediction, we performed a binding pocket detection using the DoGSiteScorer, a webserver tool (https://proteins.plus/help/dogsite) to identify potential binding pockets and assign druggability scores, which refers to the ability of a target to be functionally modulated by a small drug-like molecule^67^. In DoGsiteScorer the druggability score is computed using a linear combination of pocket descriptors such as volume, hydrophobicity, and enclosure. This server also calculates properties, describing size, shape, and chemical features of the predicted pockets^67^.

## Results

### *Sm*TGR expression

cDNA encoding *Sm*TGR was cloned from a cDNA prepared from the schistosomes and the native sequence (residues 2–596), produced as an N-terminal SUMO fusion in insect cells using the baculovirus expression system (Suppl. Mat. Figure S1). The protein was purified from lysed cells by a combination of immobilized metal affinity chromatography (IMAC) and size exclusion chromatography (SEC) (Suppl. Mat. Figure S2). The SUMO fusion protein was cleaved by S3C protease digestion followed by reverse IMAC and SEC. The purified *Sm*TGR protein had molecular weight of 65 kDa, with a purity of ≥ 90% by SDS–polyacrylamide gel electrophoresis.

### *Sm*TGR apo crystal structure

**Figure 1:**
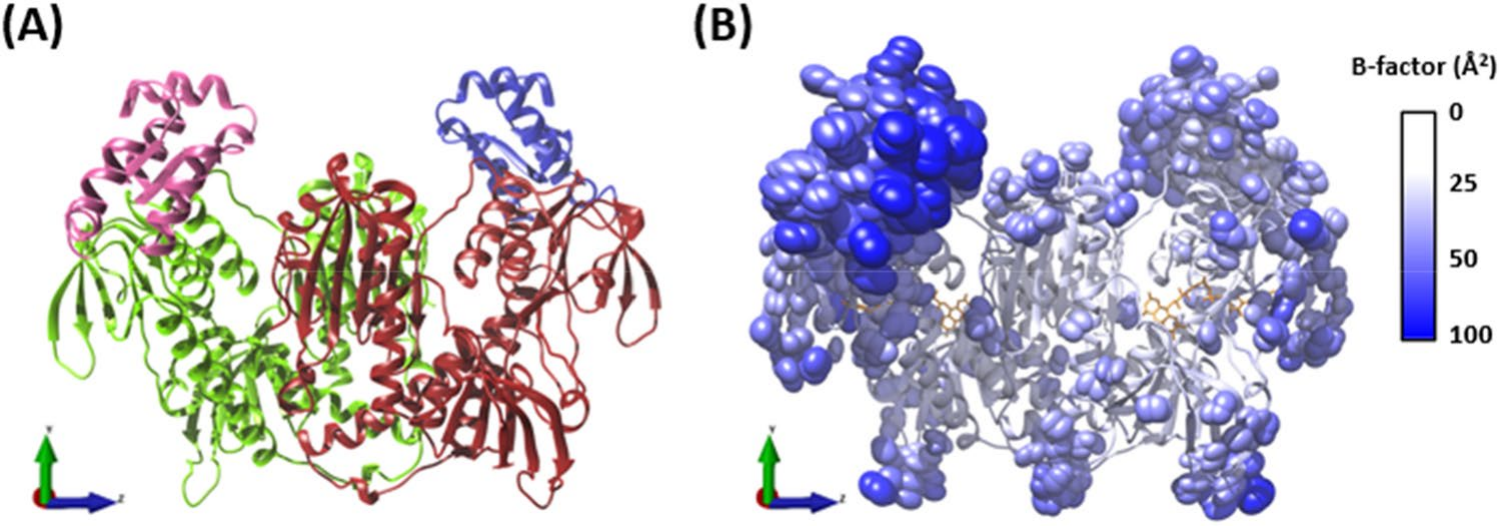
Ribbon structure of *Sm*TGR. (**A**) *Sm*TGR biological assembly containing two monomers, each composed of both Grx and TrxR domains. The colors on the left side depict Grx (pink) and TrxR (green) domains at subunit A. The colors on the right side depict Grx (blue) and TrxR (red) domains at subunit B. (**B**) The side chain atoms with values above 30 Å^2^ are depicted as anisotropic ellipsoids superposed on *Sm*TGR ribbon backbone. Both ribbon representation of backbone and side-chains ellipsoids are colored according to the *B*-factor color scale. FAD cofactor is represented in orange sticks. These images were generated with UCSF Chimera 1.17.3 (build 42,480). The 3D coordinate axes were manually included.

Concentrated *Sm*TGR (12 mg/mL) was dispensed into crystallization wells containing different formulations of crystallization buffers. A solution containing 0.1 M tris pH 8.5, 0.2 M magnesium chloride and 15% w/v polyethylene glycol 4000 was able to initiate the formation of crystals (Suppl. Mat. Figure S3). Both the protein in solution and crystal were yellow since the FAD group attached to the enzyme was in fully oxidized state. This crystal was collected and entered to Diamond beamline to be X-ray diffracted. A high-resolution dataset was collected at the beamline I04-1 at 100 K and processed with the fully automated pipelines at Diamond Light Source^49^. The data obtained shows a crystalline form in the P 1 21 1 space group that allowed the generation of a 3D atomic model of the enzyme with 1.25 Å resolution (Fig. 1A). Due to the high quality and resolution of the data, anisotropic *B*-factor refinement was performed (Fig. 1B). Data collection statistics are given in Table S1, and the structure has been deposited with PDB id 8PDD. Overall, the structure is identical to those previously reported^28,68^ and comprises a homodimer in the asymmetric unit with residues 6 to 593 resolved in each monomer. The monomers are formed from a fusion of two domains Grx (1–106) and TrxR (107–593) with an FAD molecule in an extended conformation, bound at the interface between each TrxR domain (Fig. 1A).

### Fragment binding site analysis

**Figure 2:**
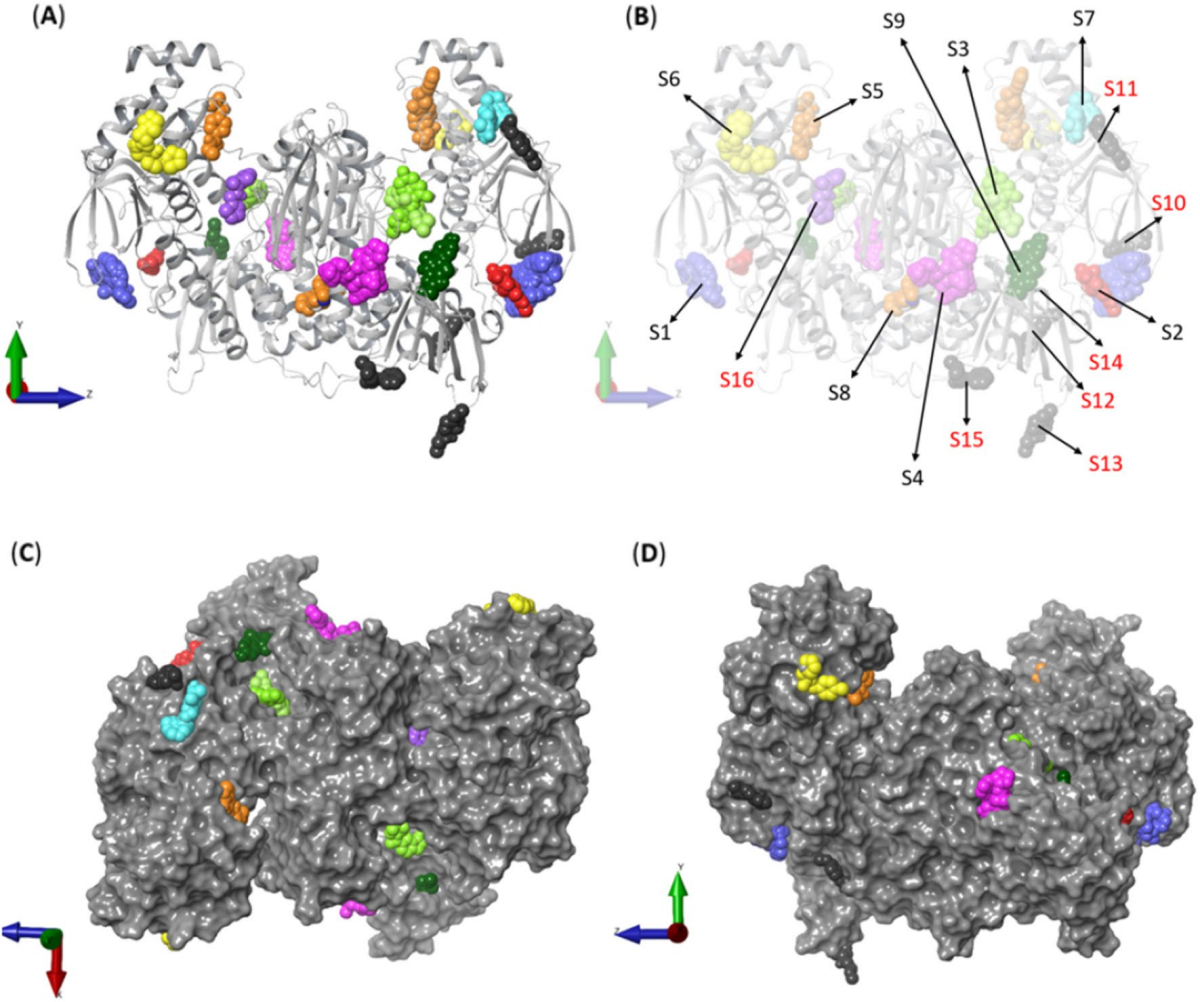
Ribbon (**A** and **B**) and Van der Waals surface (VdW; **C, D**) representations of the biological assembly of *Sm*TGR with clustered fragments. The VdW surface pictures are rotated 90° vertically (**C** and **D**). Each fragment cluster is represented as colored spheres. Since some clusters were modelled in different monomers of *Sm*TGR, many of them are shown duplicated over the surface. Binding sites are indicated by arrows in (**B**) with red labels indicating the sites with a single fragment bound.

Among the 768 fragments used in the crystallography-based screening, a set of 35 fragments were identified as hits. The PDB IDs, as well as a summary of the crystallographic data collection parameters can be found in Supplementary Information (Suppl. Mat. Table S1). The whole dataset corresponds to a total of 49 binding events on 16 different sites (Fig. 2). To distinguish the sites, they were numbered S1 to S16 (Table 1). Sites S1–S9 were observed having multiple (> 1) chemotypes bound, while sites S10–S16 bound to a single fragment each. Residue composition of fragment binding sites is listed in supplementary material (Suppl. Mat. Table S2).

**Table 1:** Binding sites information summary.

Site	Number of fragments	Domain	Subdomain	Fragments
S1	14	TrxR	FAD	x2058, x2077, x2082, x2098, x2146, x2242, x2258, x2265, x2350, x2352, x2361, x2387, x2442 and x2456
S2	2	TrxR	FAD/NADPH	x2156 and x2285
S3	3	TrxR	FAD/NADPH	x2053, x2077 and x2361
S4	9	TrxR	NADPH	x2122, x2211, x2242, x2306, x2350, x2351, x2353, x2361 and x2439
S5	3	TrxR/Grx	FAD	x2082, x2156 and x2382
S6	2	Grx		x2149 and x2069
S7	3	TrxR	FAD	x2132, x2172 and x2305
S8	3	TrxR/Interface	FAD/NADPH	x2343, x2442 and x2457
S9	3	TrxR	FAD/NAPH	x2058, x2137 and x2265
S10	1	TrxR	FAD	x2098
S11	1	TrxR	FAD	x2262
S12	1	TrxR	FAD/NADPH	x2267
S13	1	TrxR	NADPH/Insertion	x2330
S14	1	TrxR	NADPH	x2132
S15	1	TrxR	NADPH	x2387
S16	1	TrxR	FAD	x2305

**Figure 3:**
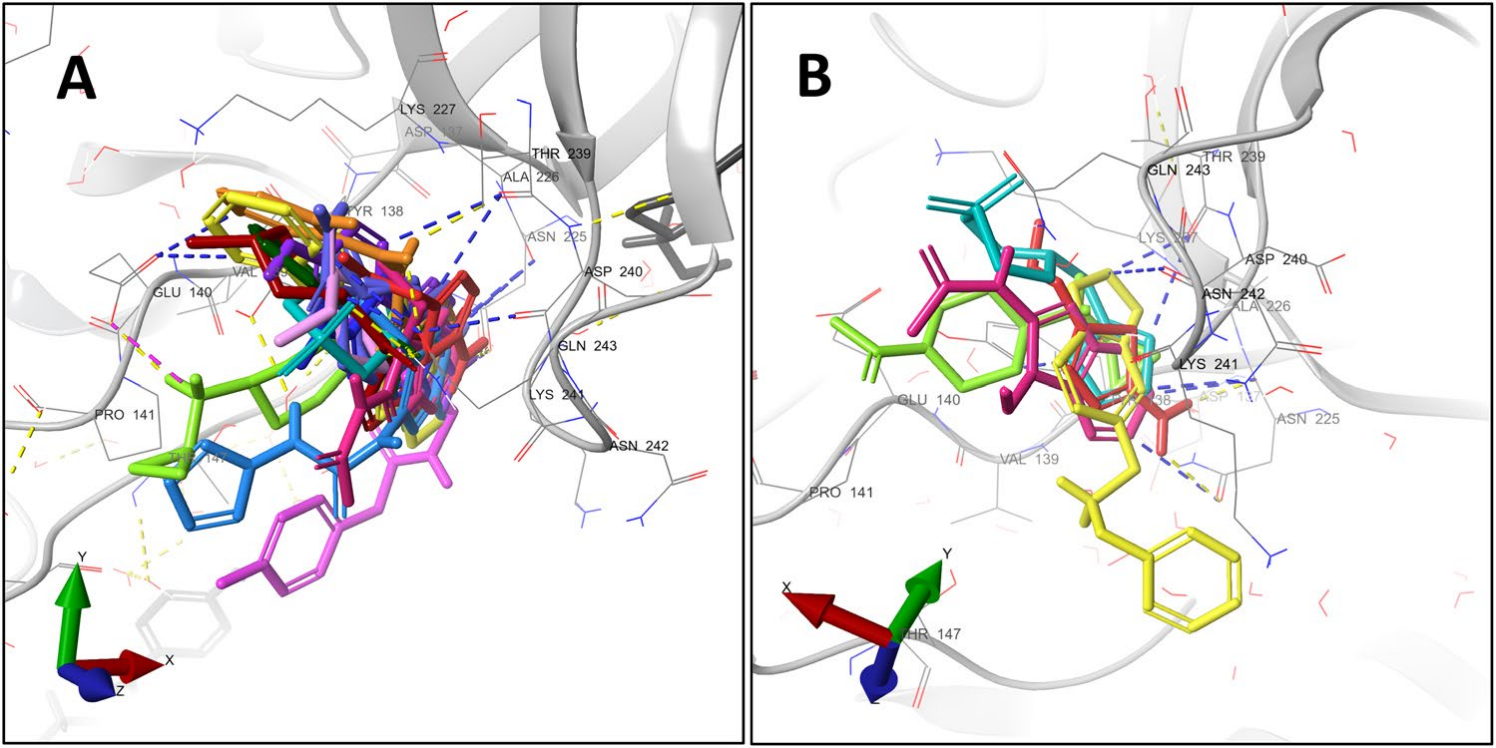
Fragment cluster at site S1. Each one of the 14 fragments is represented as thick sticks in different colors. The fragments forming this cluster were modelled in *Sm*TGR monomers A (**A**) and B (**B)**. The SmTGR is represented as a gray colored ribbon. The water molecules and protein side chains within a radius of 5 Å are represented by thin sticks in CPK colors with light gray carbons. Hydrogen bonds, π-H and salt bridges are represented by dashed yellow, blue and pink lines, respectively. Eleven fragments showed 16 π-H, while 11 hydrogen bonds were formed between *Sm*TGR and 6 fragments. The residue Asn225 makes the highest contribution to the number of polar interactions (9), through both side chain and backbone atoms.

Binding site S1 is at the TrxR domain (Fig. 3), and it is composed of residues from the FAD binding subdomain. With 14 fragments, S1 has the highest number of fragments bound. The fragments x2146, x2258, x2352, x2456 are bound exclusively to this site. Site S2 is also located at the TrxR domain, in a region between the FAD and NADPH binding subdomains. Only two fragments were observed binding at this site: x2156 and x2285 (Suppl. Mat. Figure S4).

**Figure 4:**
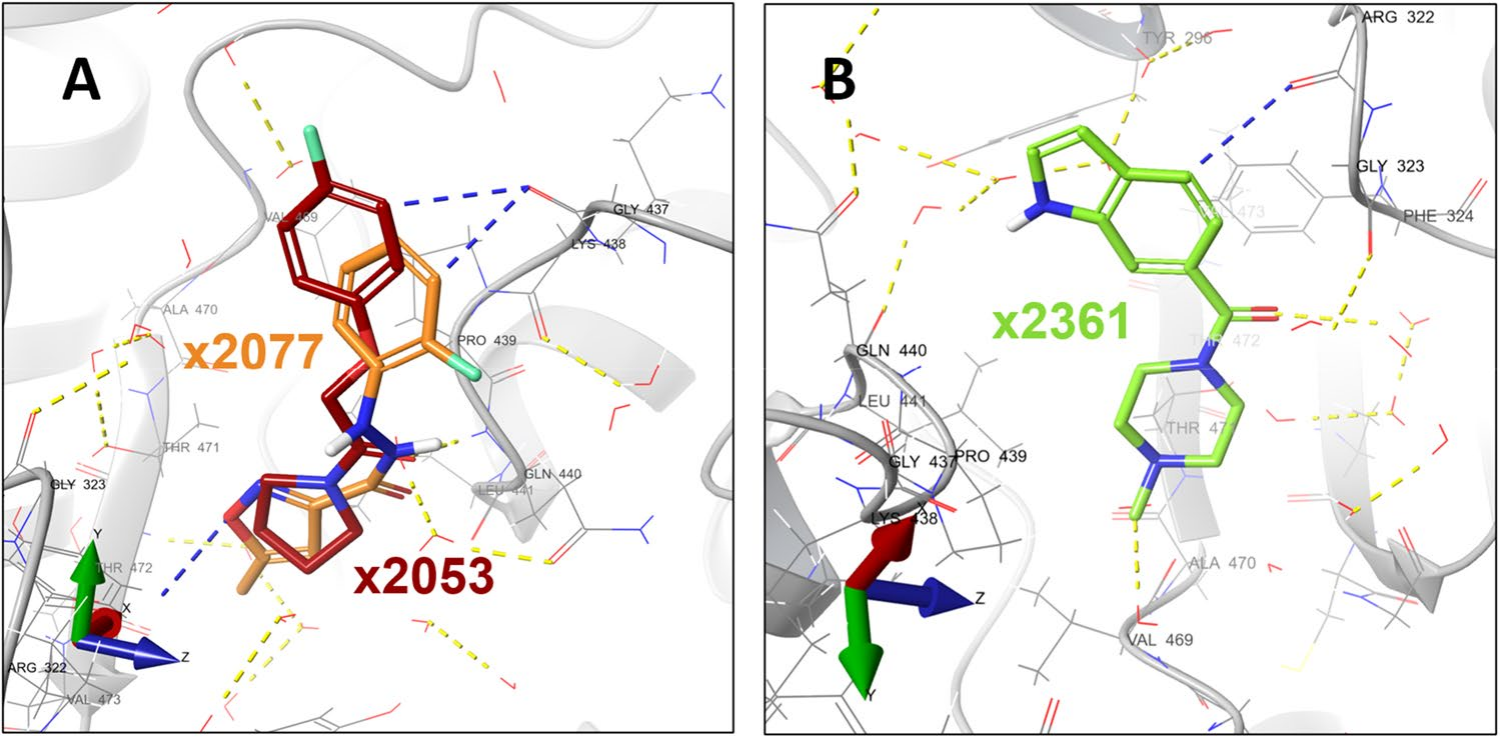
Fragments bound to binding site S3. The fragments forming this cluster were modelled in *Sm*TGR monomers A (**A**) and B (**B**). The *Sm*TGR is represented as a gray colored ribbon. The water molecules and protein side chains within a radius of 5 Å from the fragments are represented by thin sticks in CPK colors with light gray carbons. Hydrogen bonds and π-H bonds are represented by dashed yellow and blue lines, respectively. The fragments x2053 and x2361 interacted through a hydrogen bond with the residue Gln440 and a water molecule, respectively.

Site S3 includes binding events from fragments x2077, x2053 and x2361 (Fig. 4). The latter was also found bound at S1 and S4. These molecules are deeply buried in a cavity within the site. This site is also located in the FAD and NADPH binding subdomains, at the TrxR domain. This site overlaps with the coordinates of the ‘doorstop’ site previously reported by Silvestri and coworkers^69^.

Site S4 is the binding site of 9 fragments (Suppl. Mat. Figure S5). The fragments x2122, x2211, x2306, x2351, x2353 and x2439 were exclusively bound at this site. These fragments are the richest in number of interactions with the *Sm*TGR. Most of the binding events observed at this site involve aromatic rings and the residues Asp334, Phe343 and Lys345. These residues are thought to play a major role in fragment’s stabilization. Lys345 is the major residue involved in the multiple π-cation interactions observed.

Site S5 is composed of residues from the TrxR and Grx domains, lying over the FAD binding subdomain (Suppl. Mat. Figure S6). Three fragments were found at this site: x2082, x2156 and x2382. With only 7 residues, Site S6 is at the Grx domain and binds to fragments x2069 and x2149 (Suppl. Mat. Figure S7). Site S7 is at the FAD binding subdomain within TrxR domain (Suppl. Mat. Figure S8) and is comprised of 9 residues. This site spans binding events of three fragments: x2172, x2132 and x2305. The latter two were also observed at sites S14 (x2132) and S16 (x2305).

**Figure 5:**
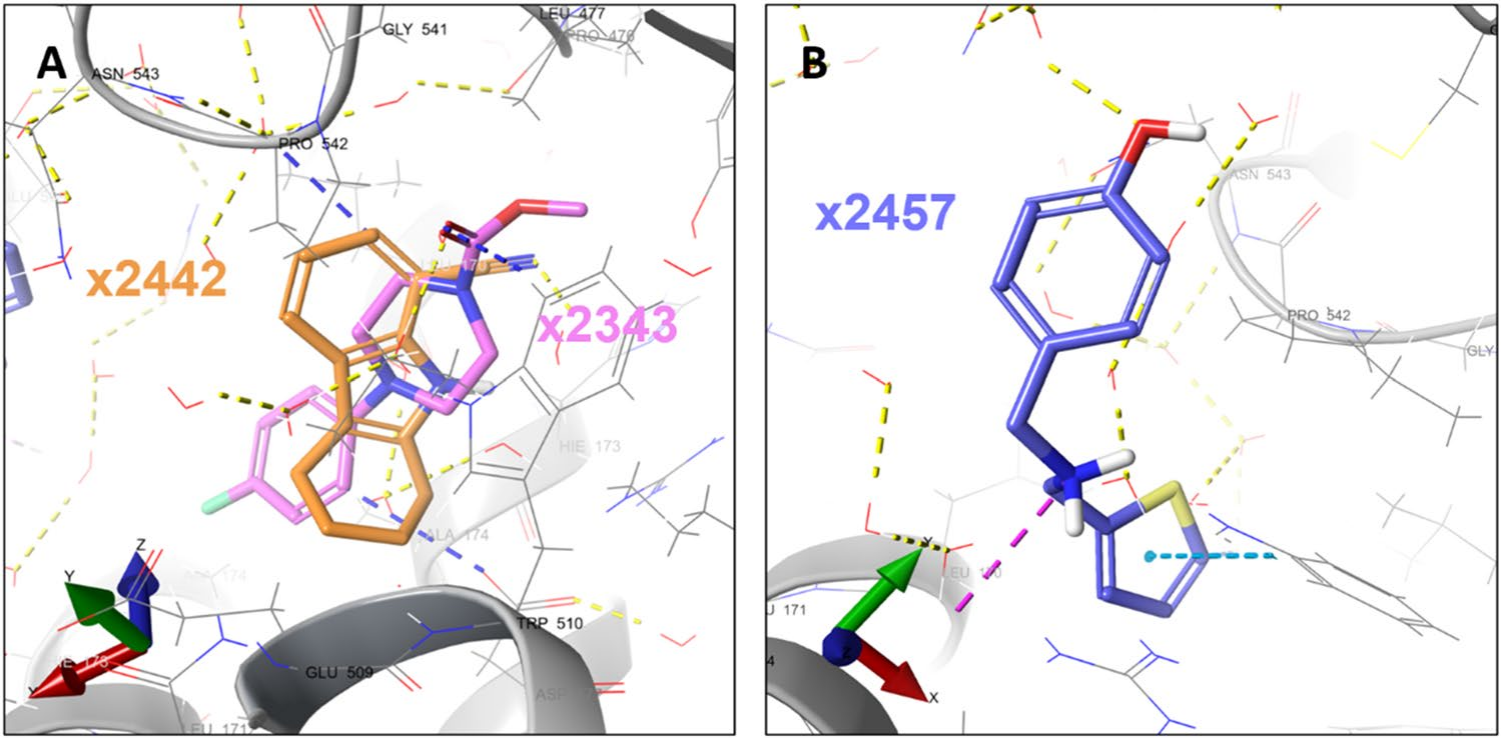
Fragments bound to site S8. (**A**) Fragments x2442 and x2343 at site S8 in chain A. (**B**) Fragment x2457 bound at site S8 in chain B. Fragments are colored in CPK with distinct carbon colors carbons, respectively. The *Sm*TGR is represented as a gray colored ribbon. The water molecules and protein side chains within a radius of 5 Å from the fragments are represented by thin sticks in CPK colors with light gray carbons. Hydrogen bonds are represented by dashed yellow lines. Interactions such as hydrogen bonds, π-π and π-H are represented by yellow, cyan, and blue dashed lines, respectively.

Site S8 spans both TrxR and interface domains. In this site fragments x2343, x2442 and x2457 are bound (Fig. 5A,B). The interface domain lies between the two polypeptide chains of the functional dimer of *Sm*TGR^28^. Residues Gln167, Leu170, Ala174 and Asn543 from both chains are part of this site. This is the largest site, with 24 residues. Except for x2442, the other two fragments were not observed elsewhere.

**Figure 6:**
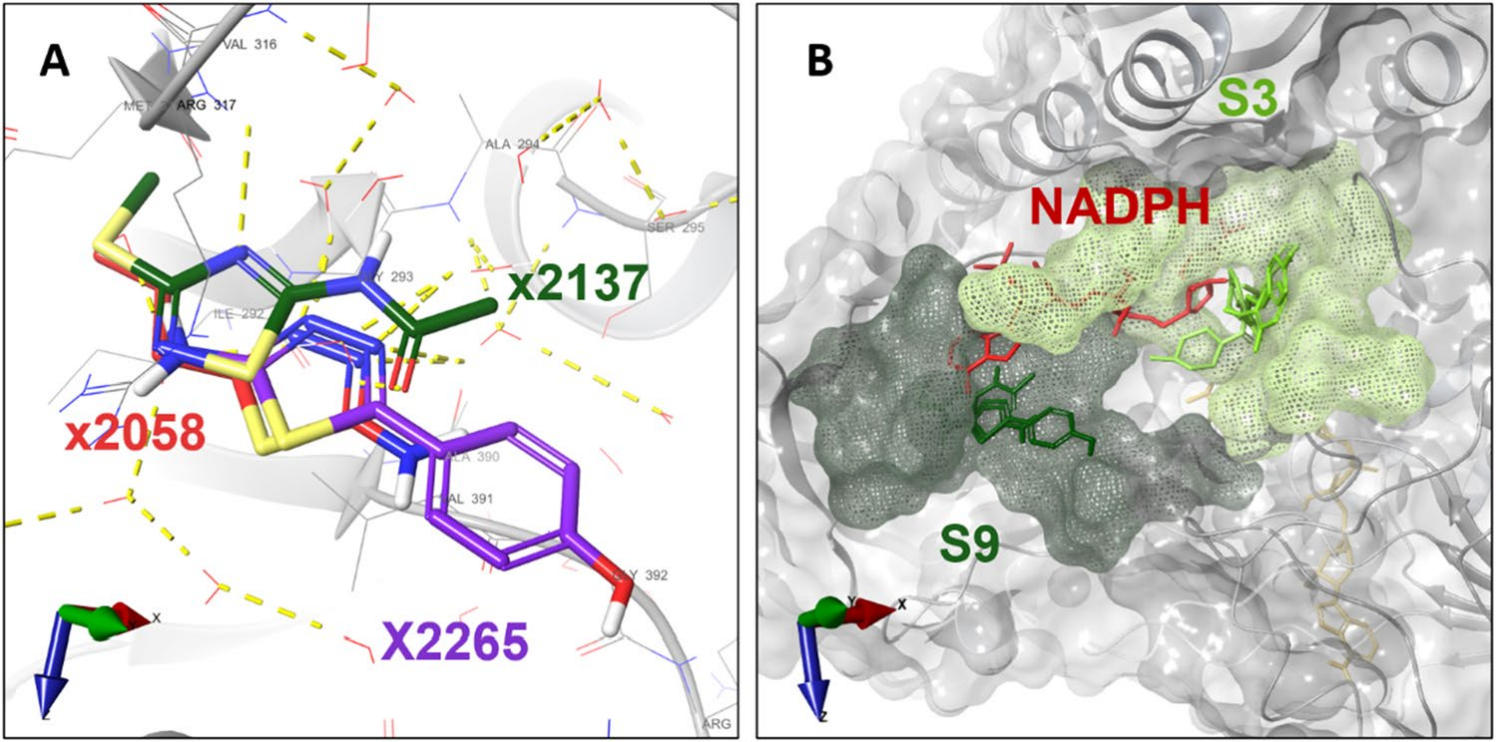
Fragments bound to site S9. (**A**) Fragments x2137, x2058 and x2265 bound to site S9. Fragments are colored in CPK with distinct carbon colors carbons, respectively. The *Sm*TGR is represented as a gray colored ribbon. The water molecules and protein side chains within a radius of 5 Å from the fragments are represented by thin sticks in CPK colors with light gray carbons. Hydrogen bonds are represented by dashed yellow lines. Interactions such as hydrogen bonds, π-π and π-H are represented by yellow, cyan, and blue dashed lines, respectively. (**B**) Proximity of sites S3 and S9 to NADPH binding site. *Sm*TGR VdW surface is shown in transparent gray with the S3 and S9 surfaces highlighted in light and dark green meshes, respectively. The NADPH coordinates were obtained from PDB entry 2X99.

Site S9 lies at the TrxR domain and is composed of residues from FAD and NAPDH binding subdomains. Fragments x2137, x2058 and x2265 were observed bound to it and are the ones most exposed to the solvent (Fig. 6A). Fragments x2058 and x2265 were also seen at site 1. Fragments x2058 and x2265 were also seen at site 1. Both S9 and S3 overlap with the NAPDH binding site (Fig. 6B).

**Figure 7:**
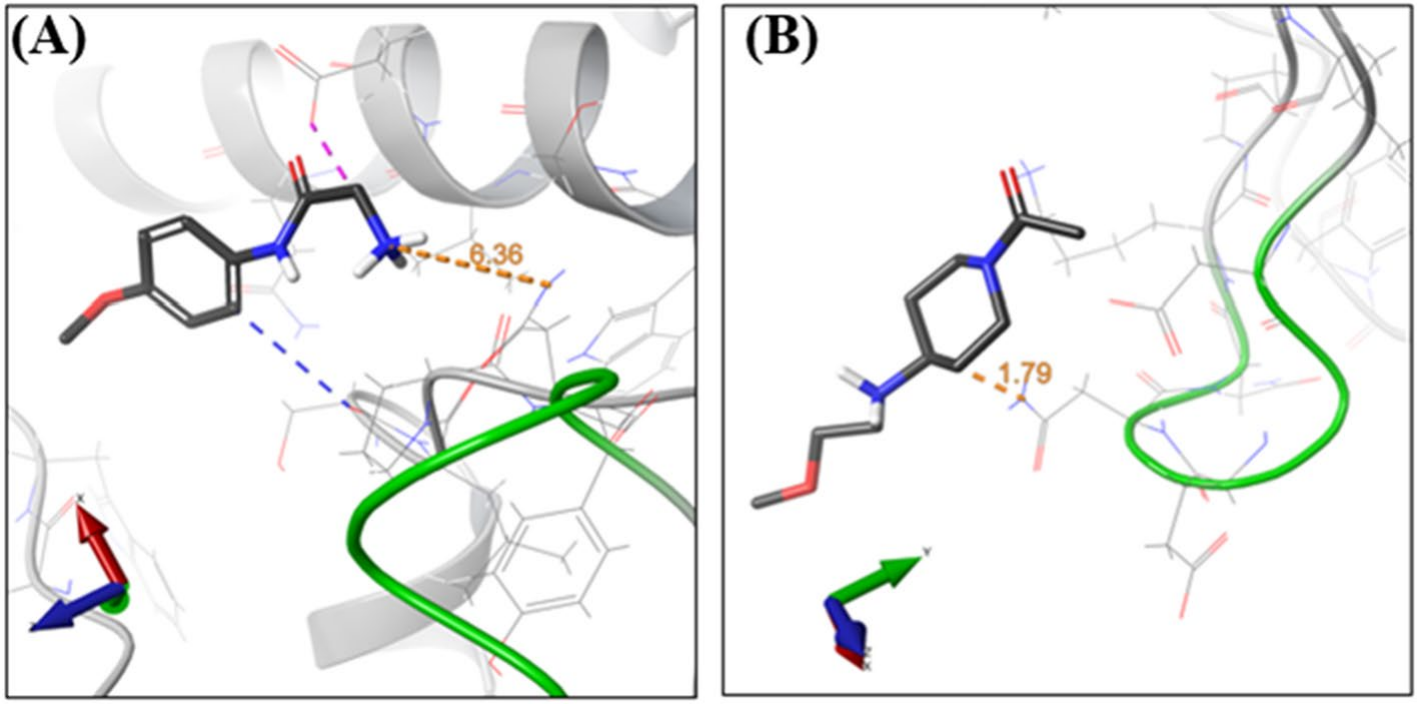
Fragments binding near the unique 359–363 insertion of SmTGR. Fragment x2267 (**A**) (S12) and fragment x2330 (**B**) (S13) interactions and its relative location to *Sm*TGR insertion (green ribbons). Atoms are colored according to the CPK scheme.

Each of the sites S10-S16 binds to a single fragment molecule (Suppl. Mat. Figure S9). One fragment to highlight is x2267, which is bound to site S12, 6.36 Å away from Asn363 residue in the insertion (359–363) (Fig. 7A), a structural feature not shared by any other homologous enzymes, and therefore, unique to SmTGR^28^. The binding site for fragment x2330 (S13) is also composed of residues from *Sm*TGR characteristic insertion and NADPH binding subdomain. Its C5 atom is 1.79 Å away from Asn362, a residue that is also part of the SmTGR unique insertion (Fig. 7B).

### Hotspots prediction

**Figure 8:**
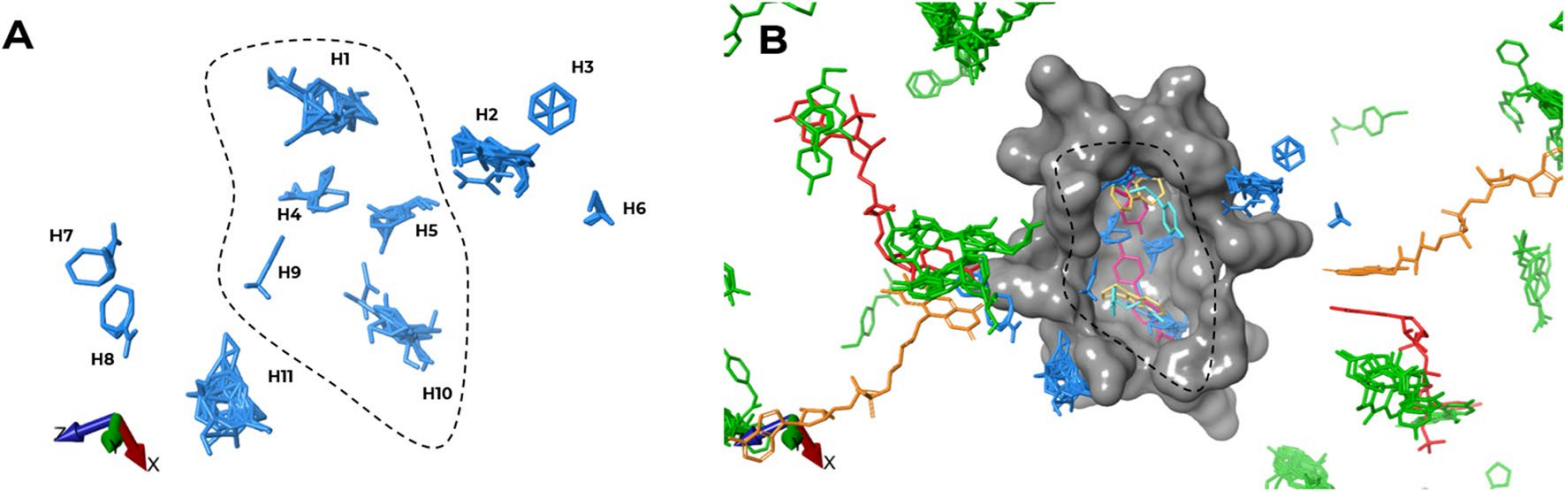
(**A**) The hotspots predicted by FTMap and their relative position. (**B**) Cartoon representation of the S8 (gray surface) highlighting the 11 hotspots (blue), FAD (orange), NADPH (red) and fragments (green). The S8 fragments (pink, yellow and cyan) can be seen inside the cavity.

The hotspot analysis identified 11 multiple-probe binding regions on the *Sm*TGR surface (Fig. 8). We assessed the relative hotspots positions and compared to *Sm*TGR structural features, cofactors, and fragment binding sites. In this section the letter H followed by a specific number was used to sequentially identify each hotspot (*e.g.*, H5). Except for S8 site, most of the hotspots did not coincide with experimental fragment binding sites. Clustering of the probe molecules occurred predominantly in S8, where 5 (H1, H4, H5, H9 and H10) out of 11 hotspots were identified. H4, H5 and H9 are in the middle of this cavity. The whole cavity spans two partially fusioned S8 sites, each formed by residues from a distinct subunit. In this scenario, H1 and H10 are equivalent hotspots in different subunits.

The 6 hotspots left (H2, H3, H6, H7, H8 and H11) were observed nearby, but not inside S8. Hotspots H7 and H8 are found between the FAD isoaloxazine moiety and the nicotinamide ring of NADPH. S3 is the closest binding site, 4.27 Å away from H7. Interesting correlations were not observed for H2, H3, H6, and H11 with respect to either the fragments or pocket identification.

### Pocket druggability analysis

Pockets found by DoGSiteScorer were labeled with the letter P followed by a specific number to sequentially identify each pocket (*e.g.*, P5). Among the 38 pockets identified, pockets P0 and P1 have the greatest internal volumes with 2601.05 Å^3^ and 2499.90 Å^3^, respectively (Suppl. Mat. Table S3). Together they form the wide cavity harboring FAD (*i.e.*, FAD binding site). The 104 residues within pocket P0 also participate in the formation of binding sites S1, S2, S3, S4, S8, S9 and S10. On the opposite chain there is P1, a pocket equivalent to P0. This cavity shares residues with S1, S2, S3, S8, S9, S10 and S12. Pockets P1 and P18 are the main cavities overlapping S9 and NADPH binding sites. According to PDB 2X99, from which NADPH coordinates were extracted, half of the NADPH molecule is accommodated in P18, as well as the residues from S9.

Consistently with the FTmap results, the pocket prediction by DoGSiteScorer identified pocket P2—third highest druggability score—spanning the whole fusioned S8 cavity (Suppl. Mat. Table S2). This pocket spans the interface region and, therefore, is composed of residues from the two subunits of *Sm*TGR. Pockets P3, P24 and P32 surround P2. Pockets P10 and P14 were predicted at chains A and B, respectively. These pockets are mutually equivalent but in opposite subunits. S8 shares residues with three of the best scored druggability pockets (P0, P1 and P2). The pockets P24 and P32 also intersect with S8.

### SmTGR inhibition assays

*Sm*TGR inhibition assays with the original fragments were limited to the 18 first hit compounds. The reason for this is that the functional assays with the fragments were performed concomitantly with the refinement of the data from the crystallography. By the time these 18 compounds were tested, only these had been confirmed as hits. None of the compounds showed more than 16.1% enzyme inhibition when tested at the concentration of 100 µM (Suppl. Mat. Figure S10).

## Discussion

*Sm*TGR crystals were previously obtained by other authors, revealing important structural characteristics of the enzyme, such as the fusion of both Grx and TrxR domains and the precise location of the huge clefts (herein defined by P0 and P1 from the DogSite analysis) that harbor the FAD molecules^28^. Another remarkable structural feature is *Sm*TGR cavity-rich topology, including a deeply buried pocket formed at the interface of both subunits (refer to S8 binding site). In this work we obtained the recombinant mutated form of the enzyme and crystallized it at 1.25 Å, a resolution higher than the previously reported highest resolution structure of 1.45 Å (PDB: 7B02). Our high-resolution *Sm*TGR crystal structure correlates with its biologically active conformation, a homodimeric protein spanning a total of 1176 residues. The missing residues on N-terminal (1–5) weren't included due to indistinct electron densities typical of terminal regions. Recently, Fata and colleagues successfully published for the first time a structure of *Sm*TGR C-terminal GCUG motif (PDB: 7B02), showing this intrinsically disordered region^68^.

The *Sm*TGR Grx domain (1–107) stands out for presenting relatively higher values of anisotropic *B*-factor, reaching up to 110.63 A^2^. This same feature could also be observed in our protein–ligand structures (not shown). Regions with *B*-factor values above 30 A^2^ can be considered highly disordered and are commonly found in both low- and high-resolution structures. This value reflects the atomic disorder, which is influenced by both static and dynamic components. In high resolution structures a relatively high *B*-factor can be mostly attributed to a dynamical disorder component (*i.e.,* protein flexibility)^70^. On the other hand, high *B*-factor values in low resolution structures can be mostly influenced by the static component (*i.e.,* crystal packing disorder)^71^. In certain conditions, the static component can also be interpreted as a sign of protein flexibility. When collecting data under cryogenic conditions, as performed in our data collection, multiple conformations of high mobility groups can be represented in crystal lattice. This can lead to atoms from specific parts of the protein having higher *B*-factors^72^. Considering the high resolution of our apo model, the relatively higher Grx domain *B*-factor can be an indicative of a high mobility of this domain.

Despite the efforts to discover *Sm*TGR inhibitors the number of publications reporting such molecules is still scarce. Some classes of compounds with activity against *Sm*TGR have been reported, such as antimony potassium tartrate, auranofin, oltipraz^23^, 8-hydroxyquinoline derivatives, oxadiazoles and naphthoquinones^73^. Most of the publications that reported the discovery of inhibitors were not accompanied by structural data^74–79^. In the absence of these data, some authors used a docking approach as an alternative to elucidate structural information about the binding sites^80,81^. In this scenario, only a few publications combine functional and structural information about the underlying mechanisms behind the inhibition of *Sm*TGR^68,69,82,83^.

In a previous study by our group^84^, a quantitative structure–activity relationship (QSAR) based virtual screen of approximately 150,000 commercial compounds was performed against *Sm*TGR using binary consensus machine learning models. With this approach, 29 compounds were prioritized and tested against the larval (schistosomula) and adult (male and female) evolutionary forms of *S. mansoni*. Among them two compounds were active on both parasites’ forms at low micromolar concentrations. Notwithstanding, these two hits also have characteristics of fragments, namely MW lower than 300 Da and less than 20 heavy atoms, being still subject to optimization. However, the lack of information about their binding site made optimization of these fragments difficult, which contrasts to the set of fragments disclosed in this work.

FBDD has the advantages of a more efficient sampling of chemical space with fewer compounds and higher hit rates^85,86^. However, fragments usually have weak binding affinities (from mM to high µM range) to the target^87,88^ and these require optimization into larger, higher-affinity compounds, a process known as fragment-to-lead (F2L) optimization. Historically, X-ray diffraction crystallography (XRD) has been seen as a time-consuming technique and, therefore, not suitable for screening libraries of compounds. This scenario is changing in recent decades, when advances in crystallization technologies and automation of beamlines have allowed a greater flow of data collection, revealing XRD as one of the most powerful tools among the biophysical methods currently in use for fragment screening^89^. Among many advantages, this technique covers important aspects which can be explored in the design of new drugs, such as the identification of exact fragment binding orientations, interacting waters around the target and protein conformational variations. In terms of sensitivity, crystallographic screening cannot be compared to any other technique, being able to identify ligands with affinities as low as sub-nanomolar and having the compound's solubility as a limit^89^. However, due to the required experimental conditions of fragment screening by XRD (where crystals are exposed to fragment molecules at very high concentrations – tens to hundreds of millimolar) there is an increased chance of observing “false positive” binding sites. A validation of these pockets observed in FBDD by XRD (with notably exception of evident functional sites that can be derived from prior enzymological knowledge) with respect to “druggability” usually requires follow-up experiments as typically carried out in F2L and medicinal chemistry studies^90^_._

In this work, the 35 crystallographic hits were systematically characterized regarding their binding site residue composition and contacts with *Sm*TGR. Unlike the protein–ligand complexes available until 2017 in PDB^91^, π-H appeared as the most frequent interaction, followed by H-bonds. Both sites S1 and S4 have the highest number of fragments as the most noticeable characteristic. Among the 16 sites, only S1, S4, S5 and S10 established π-cation contacts. Furthermore, at S4 we could observe 7 out of 9 fragments forming a π-cation each. Both the high number of π-cation interactions at site S4, as well as its high frequency of binding events draw attention to it. This suggests an important role of π-cation interactions in stabilizing the fragments at this site. Although π-cation mostly occurs between charged side chains of arginine and lysine residues^92^, at S5 the fragment x2082 is the one which provides the charged group to interact with Trp10. These interactions are frequent in structural data of enzyme-ligand complexes^91,92^. This is known as one of the strongest polar interactions, as well as one of the most common forces involved in protein–ligand complexes formation^93,94^.

Although site S1 is the biggest in number of fragments, these molecules do not show any consistent polar interactions with *Sm*TGR. Most of the polar interactions are π-H (17) and H-bond (10) bonds distributed between residues Gln243, Asp240, Thr239, Glu140, Tyr138 and Asn225. Despite the high number of binding events observed at sites S1 and S4, adding to the presence of energetically important interactions such as π-cation, neither site S1 nor S4 seems to possess features interesting enough to set them as priority in optimization.

None of the tested fragments showed more than 16.1% enzyme inhibition when tested at the concentration of 100 µM. This result is expected for fragments, which usually have weak binding affinities^87,88^. However, hit fragments have a higher ligand efficiency per atom, when compared to drug-like compounds, being good starting points for drug discovery (Hubbard & Murray, 2011).

In 2007, a quantitative high-throughput screen was performed targeting both *Sm*TGR and peroxiredoxin 2 (Prx2)^79^. From this work, due to reported difficulties in crystallization and structure determination of complexes between *Sm*TGR and high-affinity ligands directed to the enzyme active site, Silvestri and coworkers^69^ rescued two fragment-like compounds (1,8-naphthyridine-2-carboxylate and 1-(2-hydroxyethyl)piperazine). These fragments were capable of binding to a secondary pocket, at different subsites adjacent to the NADPH binding site of *Sm*TGR and inhibit *Sm*TGR by a competitive mechanism with NADPH. They named this binding site as "doorstop pocket" due to its inhibition mechanism. In the presence of NADPH, the Tyr296 side chain of *Sm*TGR adopts an open conformation, allowing the approximation of nicotinamide moiety of NADPH to FAD isoalloxazine ring. This Tyr296 open conformation sterically prevents the inhibitor binding, even though both NADPH and the inhibitor sites are not spatially overlapped. By contrast, the presence of the inhibitor forces the Tyr296 side chain to a closed conformation, hindering the NADPH attachment, and leading to enzyme inhibition. The fragments bound to site S3 occupied the same subsite as the fragments described at the "doorstop pocket". Together with site S9, these sites span, with no overlaps, the whole NADPH binding site. Recently, non-covalent inhibitors of SmTGR, presenting antischistosomal efficacy in an animal model of infection, were developed targeting the doorstop pocket (here site S3), which further validates this pocket as a druggable site^96^.

A literature search allowed us to identify two human glutathione reductase (*Hs*GR) inhibitors^97,98^ with structural data available in PDB, namely xanthene (1XAN) and pyocyanin (3SQP). A comparative structural analysis identified these inhibitors within site S8 coordinates. *Sm*TGR and *Hs*GR show similar functions in redox metabolism^23^ and share a similar fold. A local alignment using the BLAST server showed 37% identity among 482 residues aligned between *Sm*TGR (UniProtKB: Q962Y6) and *Hs*GR (UniProtKB: P00390). Among the 17 residues comprising site S8, only Asp177, Pro507, Arg515 and Gly541 were identical, resulting in an identity of 23.5%. This suggests that although *Sm*TGR and *Hs*GR share homologous domains, the low identity of the region corresponding to S8 can be exploited for the development of selective inhibitors.

Among the 16 sites, only S3 and S8 had any correspondence with hotspot mapping. This indicates that fragments in these sites have adequate features to molecular recognition, representing promising starting points to be prioritized in optimization phase. Furthermore, the location of S8 intersects with hotspots H3, H9 and H10. Except for H9, all of them overlapped with the fragments of this site.

In the initial FTMap publication, the authors propose that hotspots can be distinguished from other regions of the protein based on their concave topology and the presence of a mosaic-like pattern formed by hydrophobic and polar groups^99^. Similar to the criteria incorporated in the DoGsite druggability score function, these descriptors are pivotal in the identification of hotspots^67,100^. Furthermore, the algorithm generates probe positions from rigid body docking, which may limit the interpretation of results obtained from regions of the protein with greater mobility. This provides a reasonable explanation for the limited correlation between hotspots and superficial sites, as exemplified by S1 and S4. Despite the substantial number of ligands in these sites, no hotspots were observed. On the other hand, deeper sites such as S8 and S9 showed good correlation. These are buried sites with a high degree of conformational restriction among the residues. In these cases, rigid docking may exhibit a better correlation with the experimental data.

The lack of correlation between the predicted druggable sites using DoGsite scorer and fragment binding sites can be partially attributed to the parameters employed by the scoring algorithm. Despite there is no consensus on regarding the features a binding site must have to be druggable, some authors may argue that features like hydrophobicity, shape, size, or even the combination of them are pivotal to make a site druggable^101–103^. The DoGSite scoring function incorporates geometric and physicochemical descriptors. It's worth noting that among these descriptors, volume and depth were not subjected to site size normalization^67^. Fragments are smaller, therefore, they can bind in regions of lesser depth and internal volume. In fact, in our dataset many fragments were partially exposed to the solvent. Hence, the presence of a site displaying spatial characteristics that differ from those of typical drug-like molecules could have had a detrimental effect on the druggability score. While this limitation exists, this shouldn’t be taken as a flaw in the experimental design, since this method is suitable to support FBBD campaigns^100^. This can be seen in our data set where P2, with its high druggability score, aligns with S8, where hotspots H1, H4, H5, H9, and H10 were also observed.

## Conclusions

We reported results from an unprecedent screening of a library of 768 fragments against *Sm*TGR using a semi-automated X-ray crystallography facility. We observed 49 binding events involving 35 distinct molecular fragments. These fragments were found distributed in 16 sites on *Sm*TGR molecular surface, 9 of which accommodated more than one fragment hit whereas 7 were seen binding individual ligands. Six fragments (x2053, x2058, x2077, x2137, x2265 and x2361) were found bound near the NADPH substrate binding site, including a previously described allosteric site called the “doorstop pocket”. The other three fragments were bound at a cryptic site deeply buried in the SmTGR dimer interface, identified here as site S8, which correlates well with hotspots and pocket druggability predictions. The integrated analysis of the experimental X-ray crystallography data herein presented with computational analysis and literature data on homologous enzymes strongly supports the identification of at least two druggable sites on SmTGR, i.e., sites S3 (‘doorstop’) and S8, the later described for the first time here. These sites are high-priority targets for fragment optimization strategies. Other sites may be of interest for further exploration, such as S1 (the site presenting highest number of bound fragments), S9 (for linking with fragments bound at S3) and S12/S13 (which locates at a loop exclusive to the parasite enzyme).

### Supplementary Information

Supplementary Information.

## Data Availability

The data that support the findings of this study are openly available in Protein Data Bank (PDB) at https://www.rcsb.org/, PDB ID: 8PDD, 8PL0, 8PL1, 8PL2, 8PL3, 8PL4, 8PL5, 8PL6, 8PL7, 8PL8, 8PL9, 8PLA, 8PLB, 8PLC, 8PLD, 8PLE, 8PLF, 8PLG, 8PLH, 8PLI, 8PLJ, 8PLK, 8PLL, 8PLM, 8PLN, 8PLO, 8PLP, 8PLQ, 8PLR, 8PLS, 8PLT, 8PLU, 8PLV, 8PLW, 8PLX, 8PLY.
