# Supplementary material for: Fragment library screening by X-ray crystallography and binding site analysis on thioredoxin glutathione reductase of Schistosoma mansoni
Source: Sci Rep. 2024 Jan 18;14:1582. doi: 10.1038/s41598-024-52018-2 (PMC10796382; doi:10.1038/s41598-024-52018-2)
Supplement: Supplementary file 1 — Supplementary Information. [file 41598_2024_52018_MOESM1_ESM.docx]

# **SUPPLEMENTARY MATERIAL**

**Fragment library screening by X-ray crystallography and binding site analysis on thioredoxin glutathione reductase of *Schistosoma mansoni***

Lauro Ribeiro de Souza Neto^1ǂ^, Bogar Omar Montoya^1ǂ^, José Teófilo Moreira-Filho^2ǂ^, José Brandão-Neto^7,8^, Anil Verma^5^, Sebastian Bowyer^6^, Rafael Ferreira Dantas^1^, Bruno Junior Neves^3^, Frank von Delft^7,8,9,10^, Raymond J. Owens^4,5^*, Nicholas Furnham^6^*, Carolina Horta Andrade^2,11^*, Floriano Paes Silva-Jr^1^*

**Table S1** | Crystallographic parameters of data collection and statistics of refinement by fragment.

| **Fragment** |  | **x2053** | **x2058** | **x2069** | **x2077** | **x2082** | **x2098** |
| --- | --- | --- | --- | --- | --- | --- | --- |
| **PDB ID** | 8PDD | 8PL0 | 8PL1 | 8PL2 | 8PL3 | 8PL4 | 8PL5 |
| **Hit Number** |  | 1 | 2 | 3 | 4 | 5 | 6 |
| **Wavelength** | 0.97626 | 0.92819 | 0.92819 | 0.92819 | 0.92819 | 0.92819 | 0.92819 |
| **Resolution range** | 61.85 - 1.25 (1.295 - 1.25) | 29.67 - 1.7 (1.761 - 1.7) | 57.47 - 2.46 (2.548 - 2.46) | 66.04 - 1.88 (1.947 - 1.88) | 57.27 - 1.65 (1.709 - 1.65) | 57.27 - 1.97 (2.04 - 1.97) | 62.5 - 2.15 (2.227 - 2.15) |
| **Space group** | P 1 21 1 | P 1 21 1 | P 1 21 1 | P 1 21 1 | P 1 21 1 | P 1 21 1 | P 1 21 1 |
| **Unit cell** | 61.9 102.4 131.46 90 92.39 90 | 62.626 103.919 134.14 90 91.975 90 | 62.57 104.04 135.25 90 91.86 90 | 62.49 103.41 132.21 90 92.62 90 | 62.42 103.41 134.22 90 91.81 90 | 62.35 103.37 133.73 90 92.06 90 | 62.544 103.623 133.76 90 92.172 90 |
| **Total reflections** | 2964675 (249539) | 353982 (34425) | 116170 (11535) | 260864 (26403) | 390830 (39194) | 227846 (22587) | 174186 (16758) |
| **Unique reflections** | 450259 (44965) | 185712 (18391) | 61995 (6121) | 135955 (13591) | 203600 (20278) | 119349 (11859) | 92954 (9202) |
| **Multiplicity** | 6.6 (5.5) | 1.9 (1.9) | 1.9 (1.9) | 1.9 (1.9) | 1.9 (1.9) | 1.9 (1.9) | 1.9 (1.8) |
| **Completeness (%)** | 99.93 (99.96) | 98.60 (98.16) | 98.23 (97.31) | 99.69 (99.98) | 99.61 (99.90) | 99.55 (99.80) | 99.10 (99.62) |
| **Mean I/sigma(I)** | 13.71 (1.62) | 10.53 (1.50) | 7.92 (1.49) | 8.45 (1.51) | 8.31 (1.21) | 6.62 (1.23) | 7.85 (2.09) |
| **Wilson B-factor** | 14.51 | 23.69 | 37.13 | 19.41 | 19.35 | 23 | 28.55 |
| **R-merge** | 0.05989 (1.078) | 0.03515 (0.3893) | 0.1244 (0.6438) | 0.07922 (0.5675) | 0.05719 (0.6503) | 0.08447 (0.6607) | 0.06005 (0.3691) |
| **R-meas** | 0.06499 (1.191) | 0.04971 (0.5505) | 0.176 (0.9104) | 0.112 (0.8025) | 0.08088 (0.9196) | 0.1195 (0.9344) | 0.08493 (0.522) |
| **R-pim** | 0.02498 (0.4981) | 0.03515 (0.3893) | 0.1244 (0.6438) | 0.07922 (0.5675) | 0.05719 (0.6503) | 0.08447 (0.6607) | 0.06005 (0.3691) |
| **CC1/2** | 0.999 (0.544) | 0.999 (0.722) | 0.958 (0.504) | 0.994 (0.523) | 0.997 (0.507) | 0.996 (0.529) | 0.995 (0.772) |
| **CC*** | 1 (0.839) | 1 (0.916) | 0.989 (0.819) | 0.999 (0.828) | 0.999 (0.82) | 0.999 (0.832) | 0.999 (0.933) |
| **Reflections used in refinement** | 450219 (44965) | 185569 (18391) | 61891 (6118) | 135911 (13591) | 203483 (20278) | 119137 (11859) | 91866 (9202) |
| **Reflections used for R-free** | 22369 (2206) | 9267 (916) | 3190 (335) | 6687 (632) | 10055 (1035) | 5882 (523) | 4652 (461) |
| **R-work** | 0.1430 (0.2465) | 0.1888 (0.2761) | 0.1939 (0.2898) | 0.1905 (0.2646) | 0.1933 (0.2983) | 0.2098 (0.3026) | 0.1982 (0.2763) |
| **R-free** | 0.1628 (0.2684) | 0.2124 (0.2992) | 0.2489 (0.3505) | 0.2200 (0.2929) | 0.2157 (0.3195) | 0.2397 (0.3273) | 0.2344 (0.3186) |
| **CC (work)** | 0.972 (0.742) | 0.967 (0.824) | 0.956 (0.695) | 0.960 (0.762) | 0.963 (0.701) | 0.964 (0.735) | 0.954 (0.639) |
| **CC (free)** | 0.969 (0.721) | 0.957 (0.780) | 0.930 (0.549) | 0.946 (0.703) | 0.956 (0.618) | 0.957 (0.693) | 0.941 (0.563) |
| **Number of non-hydrogen atoms** | 11087 | 10223 | 9807 | 9907 | 10242 | 9917 | 9886 |
| **macromolecules** | 9492 | 9017 | 8998 | 9002 | 9006 | 9006 | 9006 |
| **ligands** | 199 | 122 | 142 | 122 | 157 | 160 | 158 |
| **solvent** | 1396 | 1084 | 667 | 783 | 1079 | 751 | 722 |
| **Protein residues** | 1176 | 1175 | 1176 | 1176 | 1176 | 1176 | 1176 |
| **RMS (bonds)** | 0.006 | 0.022 | 0.008 | 0.056 | 0.056 | 0.081 | 0.088 |
| **RMS (angles)** | 0.9 | 0.99 | 0.95 | 1.38 | 1.58 | 1.78 | 3.15 |
| **Ramachandran favored (%)** | 97.6 | 97.35 | 96.84 | 97.44 | 97.1 | 96.84 | 97.18 |
| **Ramachandran allowed (%)** | 2.4 | 2.65 | 3.16 | 2.39 | 2.9 | 3.16 | 2.82 |
| **Ramachandran outliers (%)** | 0 | 0 | 0 | 0.17 | 0 | 0 | 0 |
| **Rotamer outliers (%)** | 2.16 | 0.72 | 2.56 | 0.41 | 0.61 | 0.51 | 1.33 |
| **Clashscore** | 2.35 | 1.86 | 3.07 | 1.37 | 1.53 | 1.31 | 1.64 |
| **Average B-factor** | 25.11 | 29.62 | 41.05 | 23.03 | 25.71 | 28.25 | 34.97 |
| **macromolecules** | 22.72 | 29.01 | 41.34 | 22.75 | 25.07 | 28.07 | 34.97 |
| **ligands** | 30.89 | 26.22 | 39.81 | 26.45 | 26.94 | 33.86 | 37.46 |
| **solvent** | 40.6 | 35.09 | 37.48 | 25.77 | 30.84 | 29.27 | 34.5 |
|  |  |  |  |  |  |  |  |
| **Fragment** | **x2122** | **x2132** | **x2137** | **x2146** | **x2149** | **x2156** | **x2172** |
| **PDB ID** | 8PL6 | 8PL7 | 8PL8 | 8PL9 | 8PLA | 8PLB | 8PLC |
| **Hit Number** | 7 | 8 | 9 | 10 | 11 | 12 | 13 |
| **Wavelength** | 0.92819 | 0.92819 | 0.92819 | 0.92819 | 0.92819 | 0.92819 | 0.92819 |
| **Resolution range** | 57.15 - 1.81 (1.875 - 1.81) | 29.62 - 1.77 (1.833 - 1.77) | 29.79 - 2.35 (2.434 - 2.35) | 66.66 - 1.98 (2.051 - 1.98) | 57.46 - 1.66 (1.719 - 1.66) | 37.87 - 2.0 (2.071 - 2.0) | 55.55 - 2.34 (2.424 - 2.34) |
| **Space group** | C 1 2 1 | P 1 21 1 | C 1 2 1 | P 1 21 1 | P 1 21 1 | P 1 21 1 | P 1 21 1 |
| **Unit cell** | 144.96 103.15 62.19 90 113.22 90 | 62.386 103.815 133.743 90 92.06 90 | 144.915 103.158 61.136 90 113.117 90 | 62.27 103.39 133.39 90 91.92 90 | 62.5 103.13 131.65 90 92.72 90 | 62.28 103.38 133.33 90 92.06 90 | 62.12 103.29 133.61 90 92.03 90 |
| **Total reflections** | 143400 (14527) | 318512 (31731) | 65281 (6511) | 221445 (21886) | 374197 (37458) | 212779 (21562) | 132418 (12839) |
| **Unique reflections** | 75910 (7596) | 164978 (16435) | 34053 (3347) | 116452 (11549) | 195559 (19541) | 112267 (11322) | 70234 (6959) |
| **Multiplicity** | 1.9 (1.9) | 1.9 (1.9) | 1.9 (1.9) | 1.9 (1.9) | 1.9 (1.9) | 1.9 (1.9) | 1.9 (1.8) |
| **Completeness (%)** | 99.26 (99.70) | 99.55 (99.47) | 98.55 (98.07) | 99.01 (98.67) | 99.54 (99.81) | 98.38 (99.77) | 98.16 (98.29) |
| **Mean I/sigma(I)** | 9.31 (1.35) | 11.59 (1.53) | 9.05 (1.51) | 5.10 (1.07) | 10.97 (1.41) | 6.79 (1.33) | 4.34 (1.28) |
| **Wilson B-factor** | 22.2 | 23.62 | 36.88 | 24.03 | 19.26 | 19.73 | 23.61 |
| **R-merge** | 0.08568 (0.6127) | 0.04244 (0.4995) | 0.07918 (0.5354) | 0.1149 (0.7451) | 0.04959 (0.5793) | 0.1046 (0.626) | 0.1479 (0.6509) |
| **R-meas** | 0.1212 (0.8665) | 0.06002 (0.7064) | 0.112 (0.7571) | 0.1625 (1.054) | 0.07013 (0.8193) | 0.1479 (0.8853) | 0.2091 (0.9205) |
| **R-pim** | 0.08568 (0.6127) | 0.04244 (0.4995) | 0.07918 (0.5354) | 0.1149 (0.7451) | 0.04959 (0.5793) | 0.1046 (0.626) | 0.1479 (0.6509) |
| **CC1/2** | 0.991 (0.505) | 0.999 (0.586) | 0.991 (0.654) | 0.988 (0.505) | 0.997 (0.532) | 0.989 (0.549) | 0.967 (0.633) |
| **CC*** | 0.998 (0.819) | 1 (0.859) | 0.998 (0.889) | 0.997 (0.819) | 0.999 (0.834) | 0.997 (0.842) | 0.992 (0.881) |
| **Reflections used in refinement** | 75906 (7596) | 164850 (16434) | 33994 (3347) | 116301 (11549) | 195504 (19541) | 112068 (11322) | 69903 (6959) |
| **Reflections used for R-free** | 3759 (415) | 8265 (865) | 1663 (155) | 5747 (509) | 9641 (1004) | 5560 (510) | 3475 (313) |
| **R-work** | 0.1970 (0.2851) | 0.1867 (0.2748) | 0.1989 (0.2889) | 0.2191 (0.3341) | 0.1925 (0.2842) | 0.2129 (0.2870) | 0.2269 (0.2943) |
| **R-free** | 0.2217 (0.3142) | 0.2093 (0.2899) | 0.2439 (0.3461) | 0.2553 (0.3624) | 0.2155 (0.3150) | 0.2436 (0.3134) | 0.2664 (0.3369) |
| **CC (work)** | 0.953 (0.626) | 0.966 (0.751) | 0.932 (0.476) | 0.953 (0.524) | 0.959 (0.671) | 0.963 (0.787) | 0.961 (0.807) |
| **CC (free)** | 0.949 (0.600) | 0.956 (0.751) | 0.904 (0.404) | 0.939 (0.520) | 0.952 (0.621) | 0.947 (0.783) | 0.935 (0.736) |
| **Number of non-hydrogen atoms** | 5009 | 10222 | 4784 | 9946 | 9911 | 9908 | 10110 |
| **macromolecules** | 4503 | 9006 | 4503 | 9006 | 9006 | 9006 | 9006 |
| **ligands** | 87 | 154 | 75 | 134 | 140 | 154 | 130 |
| **solvent** | 419 | 1062 | 206 | 806 | 765 | 748 | 974 |
| **Protein residues** | 588 | 1176 | 588 | 1176 | 1176 | 1176 | 1176 |
| **RMS (bonds)** | 0.031 | 0.036 | 0.008 | 0.026 | 0.043 | 0.008 | 0.035 |
| **RMS (angles)** | 1.38 | 1.66 | 0.93 | 1.3 | 1.79 | 0.96 | 1.63 |
| **Ramachandran favored (%)** | 97.61 | 97.18 | 96.59 | 96.76 | 97.53 | 97.1 | 95.9 |
| **Ramachandran allowed (%)** | 2.39 | 2.82 | 3.41 | 3.24 | 2.47 | 2.9 | 4.1 |
| **Ramachandran outliers (%)** | 0 | 0 | 0 | 0 | 0 | 0 | 0 |
| **Rotamer outliers (%)** | 0.61 | 0.41 | 1.64 | 0.72 | 0.41 | 1.12 | 1.64 |
| **Clashscore** | 1.64 | 2.3 | 2.63 | 1.75 | 0.93 | 1.53 | 2.96 |
| **Average B-factor** | 26.47 | 30.22 | 40.45 | 29.66 | 24.03 | 23.27 | 27.43 |
| **macromolecules** | 26.09 | 29.68 | 40.5 | 29.55 | 23.63 | 23.1 | 27.33 |
| **ligands** | 30.5 | 31.7 | 42.55 | 31.44 | 30.59 | 25.99 | 24.42 |
| **solvent** | 29.68 | 34.64 | 38.55 | 30.59 | 27.51 | 24.76 | 28.84 |
|  |  |  |  |  |  |  |  |
| **Fragment** | **x2211** | **x2242** | **x2258** | **x2262** | **x2265** | **x2267** | **x2285** |
| **PDB ID** | 8PLD | 8PLE | 8PLF | 8PLG | 8PLH | 8PLI | 8PLJ |
| **Hit Number** | 14 | 15 | 16 | 17 | 18 | 19 | 20 |
| **Wavelength** | 0.92819 | 0.92819 | 0.92819 | 0.92819 | 0.92819 | 0.92819 | 0.92819 |
| **Resolution range** | 33.83 - 1.85 (1.916 - 1.85) | 62.20 - 1.94 (2.009 - 1.94) | 29.53 - 1.95 (2.023 - 1.95) | 29.57 - 1.75 (1.813 - 1.75) | 29.43 - 1.62 (1.678 - 1.62) | 41.16 - 2.33 (2.413 - 2.33) | 29.52 - 2.18 (2.258 - 2.18) |
| **Space group** | P 1 21 1 | P 1 21 1 | P 1 21 1 | C 1 2 1 | C 1 2 1 | P 1 21 1 | P 1 21 1 |
| **Unit cell** | 62.11 103.2 133.97 90 91.85 90 | 62.23 103.79 134.41 90 91.76 90 | 62.24 103.472 133.568 90 92.016 90 | 144.793 103.563 59.629 90 113.001 90 | 146.154 103.498 62.207 90 113.433 90 | 62.47 103.84 133.54 90 91.96 90 | 62.069 103.48 134.549 90 91.911 90 |
| **Total reflections** | 275462 (28082) | 237474 (23724) | 234928 (23096) | 152505 (14867) | 206639 (20670) | 129566 (12600) | 159885 (15377) |
| **Unique reflections** | 143472 (14328) | 124633 (12385) | 121963 (11976) | 79750 (7879) | 107317 (10717) | 70758 (6959) | 86402 (8534) |
| **Multiplicity** | 1.9 (2.0) | 1.9 (1.9) | 1.9 (1.9) | 1.9 (1.9) | 1.9 (1.9) | 1.8 (1.8) | 1.9 (1.8) |
| **Completeness (%)** | 99.65 (99.90) | 98.57 (98.40) | 99.40 (98.18) | 97.72 (96.85) | 99.68 (99.86) | 96.99 (95.64) | 97.23 (96.91) |
| **Mean I/sigma(I)** | 8.49 (1.41) | 5.80 (1.14) | 7.69 (1.54) | 11.35 (1.55) | 12.48 (1.65) | 10.57 (2.18) | 6.67 (1.13) |
| **Wilson B-factor** | 22.19 | 25.97 | 25.8 | 23.55 | 21.29 | 33.36 | 34.04 |
| **R-merge** | 0.06232 (0.5934) | 0.07617 (0.7164) | 0.06584 (0.4874) | 0.04019 (0.4664) | 0.03286 (0.4185) | 0.1605 (0.7515) | 0.08138 (0.674) |
| **R-meas** | 0.08814 (0.8391) | 0.1077 (1.013) | 0.09311 (0.6893) | 0.05684 (0.6596) | 0.04647 (0.5919) | 0.227 (1.063) | 0.1151 (0.9531) |
| **R-pim** | 0.06232 (0.5934) | 0.07617 (0.7164) | 0.06584 (0.4874) | 0.04019 (0.4664) | 0.03286 (0.4185) | 0.1605 (0.7515) | 0.08138 (0.674) |
| **CC1/2** | 0.997 (0.512) | 0.996 (0.477) | 0.997 (0.668) | 0.998 (0.578) | 0.999 (0.694) | 0.958 (0.244) | 0.995 (0.501) |
| **CC*** | 0.999 (0.823) | 0.999 (0.804) | 0.999 (0.895) | 1 (0.856) | 1 (0.905) | 0.989 (0.626) | 0.999 (0.817) |
| **Reflections used in refinement** | 143372 (14328) | 124393 (12385) | 121736 (11972) | 79582 (7879) | 107248 (10717) | 70674 (6952) | 86242 (8534) |
| **Reflections used for R-free** | 7022 (649) | 6193 (615) | 6045 (552) | 3959 (418) | 5406 (561) | 3520 (322) | 4321 (454) |
| **R-work** | 0.1968 (0.2819) | 0.2053 (0.3126) | 0.2012 (0.2734) | 0.2078 (0.2522) | 0.2184 (0.2689) | 0.2267 (0.3223) | 0.1965 (0.3001) |
| **R-free** | 0.2243 (0.3127) | 0.2353 (0.3313) | 0.2302 (0.2860) | 0.2350 (0.2667) | 0.2344 (0.2969) | 0.2886 (0.3831) | 0.2368 (0.3272) |
| **CC (work)** | 0.962 (0.724) | 0.966 (0.758) | 0.965 (0.807) | 0.950 (0.778) | 0.946 (0.735) | 0.943 (0.657) | 0.963 (0.647) |
| **CC (free)** | 0.949 (0.661) | 0.952 (0.717) | 0.949 (0.784) | 0.943 (0.727) | 0.948 (0.699) | 0.901 (0.475) | 0.942 (0.513) |
| **Number of non-hydrogen atoms** | 9919 | 10298 | 10244 | 4738 | 4763 | 10167 | 10112 |
| **macromolecules** | 8995 | 9006 | 8988 | 4483 | 4483 | 8980 | 9006 |
| **ligands** | 158 | 178 | 142 | 66 | 92 | 119 | 134 |
| **solvent** | 766 | 1114 | 1114 | 189 | 188 | 1068 | 972 |
| **Protein residues** | 1175 | 1176 | 1174 | 586 | 586 | 1174 | 1176 |
| **RMS (bonds)** | 0.009 | 0.198 | 0.074 | 0.037 | 0.035 | 0.061 |  |
| **RMS (angles)** | 0.97 | 3.89 | 3.01 | 1.57 | 2.59 | 1.85 | 0.099 |
| **Ramachandran favored (%)** | 97.35 | 97.1 | 97.17 | 97.07 | 96.9 | 95.45 | 1.85 |
| **Ramachandran allowed (%)** | 2.65 | 2.9 | 2.74 | 2.93 | 3.1 | 4.38 | 96.59 |
| **Ramachandran outliers (%)** | 0 | 0 | 0.09 | 0 | 0 | 0.17 | 3.41 |
| **Rotamer outliers (%)** | 0.51 | 0.61 | 0.82 | 0.62 | 0.21 | 3.18 | 0 |
| **Clashscore** | 1.59 | 3.23 | 2.63 | 1.65 | 1.54 | 3.3 | 1.02 |
| **Average B-factor** | 26.51 | 31.4 | 30.53 | 27.65 | 26.81 | 36.95 | 2.52 |
| **macromolecules** | 26.36 | 30.8 | 30 | 27.54 | 26.64 | 36.82 | 39.76 |
| **ligands** | 26.2 | 33.36 | 30.76 | 27.62 | 30.18 | 34.08 | 39.62 |
| **solvent** | 28.27 | 35.91 | 34.76 | 30.17 | 29.26 | 38.39 | 37.07 |
|  |  |  |  |  |  |  |  |
| **Fragment** | **x2305** | **x2306** | **x2330** | **x2343** | **x2350** | **x2351** | **x2352** |
| **PDB ID** | 8PLK | 8PLL | 8PLM | 8PLN | 8PLO | 8PLP | 8PLQ |
| **Hit Number** | 21 | 22 | 23 | 24 | 25 | 26 | 27 |
| **Wavelength** | 0.92819 | 0.92819 | 0.92819 | 0.92819 | 0.92819 | 0.92819 | 0.92819 |
| **Resolution range** | 29.49 - 1.82 (1.885 - 1.82) | 54.84 - 1.85 (1.916 - 1.85) | 57.14 - 1.95 (2.02 - 1.95) | 29.48 - 2.8 (2.9 - 2.8) | 62.11 - 1.74 (1.802 - 1.74) | 29.64 - 2.28 (2.362 - 2.28) | 29.61 - 2.291 (2.373 - 2.291) |
| **Space group** | P 1 21 1 | C 1 2 1 | C 1 2 1 | C 1 2 1 | P 1 21 1 | P 1 21 1 | P 1 21 1 |
| **Unit cell** | 62.159 103.298 133.332 90 92.221 90 | 145.18 103.63 59.61 90 113.08 90 | 145.989 103.984 62.22 90 113.317 90 | 146.56 103.571 62.254 90 113.294 90 | 62.14 103.56 134.42 90 91.82 90 | 62.103 104.095 134.786 90 91.679 90 | 62.301 103.838 134.815 90 91.598 90 |
| **Total reflections** | 286699 (28116) | 131335 (12971) | 117617 (11630) | 37879 (3770) | 334704 (33262) | 148564 (13877) | 147390 (14316) |
| **Unique reflections** | 149097 (14708) | 68740 (6805) | 61549 (6042) | 20462 (2003) | 173699 (17313) | 77296 (7314) | 76578 (7483) |
| **Multiplicity** | 1.9 (1.9) | 1.9 (1.9) | 1.9 (1.9) | 1.9 (1.9) | 1.9 (1.9) | 1.9 (1.9) | 1.9 (1.9) |
| **Completeness (%)** | 98.98 (97.90) | 99.40 (98.97) | 98.98 (98.52) | 96.66 (95.43) | 99.68 (99.84) | 98.55 (93.97) | 99.12 (97.66) |
| **Mean I/sigma(I)** | 11.22 (1.62) | 9.11 (1.53) | 12.91 (1.93) | 6.53 (1.59) | 7.03 (1.07) | 9.29 (1.49) | 10.55 (1.49) |
| **Wilson B-factor** | 25.05 | 24.75 | 28.7 | 45.29 | 22.87 | 39.04 | 41.09 |
| **R-merge** | 0.04599 (0.4794) | 0.07364 (0.5403) | 0.04085 (0.4263) | 0.1448 (0.5474) | 0.05697 (0.693) | 0.07063 (0.6402) | 0.05117 (0.5047) |
| **R-meas** | 0.06504 (0.6779) | 0.1041 (0.7641) | 0.05776 (0.6029) | 0.2047 (0.7741) | 0.08057 (0.9801) | 0.09988 (0.9053) | 0.07236 (0.7137) |
| **R-pim** | 0.04599 (0.4794) | 0.07364 (0.5403) | 0.04085 (0.4263) | 0.1448 (0.5474) | 0.05697 (0.693) | 0.07063 (0.6402) | 0.05117 (0.5047) |
| **CC1/2** | 0.998 (0.581) | 0.9 (0.523) | 0.999 (0.728) | 0.955 (0.579) | 0.997 (0.518) | 0.996 (0.681) | 0.998 (0.806) |
| **CC*** | 1 (0.857) | 0.973 (0.829) | 1 (0.918) | 0.988 (0.857) | 0.999 (0.826) | 0.999 (0.9) | 1 (0.945) |
| **Reflections used in refinement** | 149017 (14707) | 68734 (6805) | 61455 (6041) | 20433 (2003) | 173477 (17313) | 77080 (7314) | 76465 (7483) |
| **Reflections used for R-free** | 7432 (733) | 3380 (347) | 3031 (289) | 991 (91) | 8517 (880) | 3831 (372) | 3810 (386) |
| **R-work** | 0.1862 (0.2735) | 0.2106 (0.2676) | 0.2227 (0.2831) | 0.2316 (0.2788) | 0.2096 (0.3187) | 0.1885 (0.3086) | 0.1841 (0.2774) |
| **R-free** | 0.2157 (0.3060) | 0.2376 (0.2914) | 0.2502 (0.3140) | 0.3195 (0.4055) | 0.2309 (0.3460) | 0.2296 (0.3438) | 0.2273 (0.3113) |
| **CC (work)** | 0.963 (0.735) | 0.948 (0.708) | 0.937 (0.671) | 0.898 (0.419) | 0.962 (0.702) | 0.958 (0.662) | 0.957 (0.652) |
| **CC (free)** | 0.947 (0.678) | 0.938 (0.721) | 0.922 (0.615) | 0.824 (0.231) | 0.958 (0.652) | 0.936 (0.582) | 0.935 (0.496) |
| **Number of non-hydrogen atoms** | 10229 | 4754 | 4630 | 4729 | 9920 | 10044 | 10348 |
| **macromolecules** | 8988 | 4494 | 4494 | 4503 | 8978 | 9006 | 9006 |
| **ligands** | 166 | 69 | 81 | 68 | 157 | 136 | 121 |
| **solvent** | 1075 | 191 | 55 | 158 | 785 | 902 | 903 |
| **Protein residues** | 1174 | 587 | 587 | 588 | 1174 | 1176 | 1176 |
| **RMS (bonds)** | 0.119 | 0.03 | 0.085 | 0.065 | 0.06 | 0.038 | 0.024 |
| **RMS (angles)** | 1.71 | 1.47 | 3.3 | 1.87 | 2.01 | 1.46 | 1.38 |
| **Ramachandran favored (%)** | 97 | 97.43 | 97.6 | 90.44 | 96.82 | 96.42 | 96.25 |
| **Ramachandran allowed (%)** | 2.83 | 2.57 | 2.4 | 8.7 | 3.09 | 3.58 | 3.58 |
| **Ramachandran outliers (%)** | 0.17 | 0 | 0 | 0.85 | 0.09 | 0 | 0.17 |
| **Rotamer outliers (%)** | 0.92 | 0.41 | 0.41 | 5.52 | 1.03 | 1.23 | 1.64 |
| **Clashscore** | 1.92 | 1.21 | 1.87 | 11.51 | 1.43 | 3.01 | 3.89 |
| **Average B-factor** | 30.59 | 27.7 | 31.27 | 46.25 | 28.99 | 47.22 | 47.41 |
| **macromolecules** | 30.05 | 27.59 | 31.21 | 46.51 | 28.75 | 47.35 | 47.41 |
| **ligands** | 28.12 | 29.51 | 35.88 | 48.62 | 30.66 | 40.53 | 45 |
| **solvent** | 35.49 | 29.67 | 29.32 | 37.9 | 31.43 | 46.85 | 47.78 |
|  |  |  |  |  |  |  |  |
| **Fragment** | **x2353** | **x2361** | **x2382** | **x2387** | **x2439** | **x2442** | **x2456** |
| **PDB ID** | 8PLR | 8PLS | 8PLT | 8PLU | 8PLV | 8PLW | 8PLX |
| **Hit Number** | 28 | 29 | 30 | 31 | 32 | 33 | 34 |
| **Wavelength** | 0.92819 | 0.92819 | 0.92819 | 0.92819 | 0.92819 | 0.92819 | 0.92819 |
| **Resolution range** | 62.09 - 2.72 (2.817 - 2.72) | 29.58 - 1.8 (1.864 - 1.8) | 29.52 - 2.33 (2.415 - 2.33) | 56.15 - 2.04 (2.113 - 2.04) | 34.58 - 2.46 (2.548 - 2.46) | 28.04 - 1.78 (1.844 - 1.78) | 51.69 - 1.77 (1.833 - 1.77) |
| **Space group** | P 1 21 1 | P 1 21 1 | P 1 21 1 | P 1 21 1 | P 1 21 1 | P 1 21 1 | P 1 21 1 |
| **Unit cell** | 62.12 104.35 135.17 90 91.78 90 | 62.242 103.71 134.47 90 91.841 90 | 61.944 103.565 134.011 90 91.98 90 | 62.15 103.38 133.84 90 91.95 90 | 62.08 103.85 134.73 90 91.8 90 | 62.24 103.44 134.4 90 91.96 90 | 62.04 103.38 133.87 90 91.92 90 |
| **Total reflections** | 81226 (8498) | 304888 (30389) | 136967 (13707) | 200060 (20260) | 111542 (11122) | 311351 (30629) | 311865 (30881) |
| **Unique reflections** | 45094 (4573) | 157488 (15702) | 71304 (7082) | 106299 (10642) | 59309 (5753) | 162092 (16143) | 162302 (16125) |
| **Multiplicity** | 1.8 (1.9) | 1.9 (1.9) | 1.9 (1.9) | 1.9 (1.9) | 1.9 (1.9) | 1.9 (1.9) | 1.9 (1.9) |
| **Completeness (%)** | 96.80 (98.11) | 99.67 (99.78) | 98.49 (98.28) | 98.58 (99.40) | 95.21 (93.76) | 99.46 (99.73) | 98.67 (98.72) |
| **Mean I/sigma(I)** | 9.39 (2.08) | 11.02 (1.64) | 8.38 (1.42) | 5.92 (1.27) | 7.41 (0.93) | 5.90 (0.96) | 6.18 (0.93) |
| **Wilson B-factor** | 46.75 | 25.51 | 41.12 | 28.28 | 37.79 | 20.68 | 21.44 |
| **R-merge** | 0.06819 (0.4352) | 0.04119 (0.4775) | 0.06334 (0.5477) | 0.1152 (0.7952) | 0.1011 (0.9646) | 0.07581 (0.8242) | 0.06811 (0.873) |
| **R-meas** | 0.09644 (0.6154) | 0.05825 (0.6753) | 0.08957 (0.7746) | 0.1629 (1.125) | 0.1429 (1.364) | 0.1072 (1.166) | 0.09632 (1.235) |
| **R-pim** | 0.06819 (0.4352) | 0.04119 (0.4775) | 0.06334 (0.5477) | 0.1152 (0.7952) | 0.1011 (0.9646) | 0.07581 (0.8242) | 0.06811 (0.873) |
| **CC1/2** | 0.997 (0.756) | 0.999 (0.616) | 0.996 (0.607) | 0.99 (0.4) | 0.985 (0.242) | 0.985 (0.531) | 0.996 (0.484) |
| **CC*** | 0.999 (0.928) | 1 (0.873) | 0.999 (0.869) | 0.997 (0.756) | 0.996 (0.624) | 0.996 (0.833) | 0.999 (0.808) |
| **Reflections used in refinement** | 44993 (4573) | 157313 (15701) | 71120 (7079) | 106052 (10642) | 59173 (5753) | 161743 (16143) | 161922 (16125) |
| **Reflections used for R-free** | 2336 (224) | 7849 (798) | 3540 (317) | 5334 (560) | 3035 (315) | 8098 (839) | 8130 (869) |
| **R-work** | 0.1946 (0.2607) | 0.1912 (0.2937) | 0.1911 (0.2712) | 0.2206 (0.3146) | 0.2164 (0.3325) | 0.2234 (0.3597) | 0.2463 (0.4351) |
| **R-free** | 0.2785 (0.3537) | 0.2162 (0.3172) | 0.2296 (0.2927) | 0.2579 (0.3630) | 0.2802 (0.3937) | 0.2496 (0.3812) | 0.2660 (0.4573) |
| **CC (work)** | 0.949 (0.723) | 0.967 (0.804) | 0.968 (0.821) | 0.962 (0.803) | 0.944 (0.527) | 0.964 (0.777) | 0.959 (0.696) |
| **CC (free)** | 0.911 (0.529) | 0.955 (0.772) | 0.947 (0.746) | 0.940 (0.660) | 0.918 (0.482) | 0.951 (0.737) | 0.948 (0.702) |
| **Number of non-hydrogen atoms** | 9807 | 10197 | 9997 | 10267 | 9794 | 10244 | 10243 |
| **macromolecules** | 9006 | 9006 | 9006 | 8977 | 8989 | 8977 | 9006 |
| **ligands** | 118 | 142 | 122 | 178 | 142 | 157 | 138 |
| **solvent** | 683 | 1049 | 869 | 1112 | 663 | 1110 | 1099 |
| **Protein residues** | 1176 | 1176 | 1176 | 1173 | 1174 | 1173 | 1176 |
| **RMS (bonds)** | 0.015 | 0.044 | 0.055 | 0.107 | 0.052 | 0.047 | 0.033 |
| **RMS (angles)** | 0.99 | 1.37 | 1.5 | 1.93 | 1.91 | 2.03 | 1.71 |
| **Ramachandran favored (%)** | 93.52 | 97.1 | 96.25 | 96.73 | 95.03 | 96.9 | 96.93 |
| **Ramachandran allowed (%)** | 6.4 | 2.9 | 3.75 | 3.18 | 4.63 | 3.01 | 2.9 |
| **Ramachandran outliers (%)** | 0.09 | 0 | 0 | 0.09 | 0.34 | 0.09 | 0.17 |
| **Rotamer outliers (%)** | 5.01 | 0.72 | 1.43 | 1.03 | 2.87 | 0.62 | 0.51 |
| **Clashscore** | 6.09 | 1.37 | 2.52 | 2.85 | 4.39 | 2.31 | 2.19 |
| **Average B-factor** | 52.06 | 31.03 | 47.26 | 33.91 | 39.38 | 26.66 | 27.83 |
| **macromolecules** | 52.49 | 30.57 | 47.37 | 33.39 | 39.71 | 26.07 | 27.34 |
| **ligands** | 48.55 | 29.59 | 44.12 | 35.78 | 34.88 | 27.96 | 26.75 |
| **solvent** | 46.97 | 35.2 | 46.54 | 37.78 | 35.87 | 31.22 | 31.97 |
|  |  |  |  |  |  |  |  |
| **Fragment** | **x2457** |  |  |  |  |  |  |
| **PDB ID** | 8PLY |  |  |  |  |  |  |
| **Hit Number** | 35 |  |  |  |  |  |  |
| **Wavelength** | 0.92819 |  |  |  |  |  |  |
| **Resolution range** | 62.17 - 2.43 (2.517 - 2.43) |  |  |  |  |  |  |
| **Space group** | P 1 21 1 |  |  |  |  |  |  |
| **Unit cell** | 62.21 103.43 134.22 90 91.97 90 |  |  |  |  |  |  |
| **Total reflections** | 120223 (12399) |  |  |  |  |  |  |
| **Unique reflections** | 63272 (6369) |  |  |  |  |  |  |
| **Multiplicity** | 1.9 (1.9) |  |  |  |  |  |  |
| **Completeness (%)** | 98.51 (99.72) |  |  |  |  |  |  |
| **Mean I/sigma(I)** | 5.03 (1.20) |  |  |  |  |  |  |
| **Wilson B-factor** | 30.33 |  |  |  |  |  |  |
| **R-merge** | 0.1445 (0.8005) |  |  |  |  |  |  |
| **R-meas** | 0.2044 (1.132) |  |  |  |  |  |  |
| **R-pim** | 0.1445 (0.8005) |  |  |  |  |  |  |
| **CC1/2** | 0.98 (0.47) |  |  |  |  |  |  |
| **CC*** | 0.995 (0.8) |  |  |  |  |  |  |
| **Reflections used in refinement** | 63154 (6369) |  |  |  |  |  |  |
| **Reflections used for R-free** | 3241 (330) |  |  |  |  |  |  |
| **R-work** | 0.2035 (0.2575) |  |  |  |  |  |  |
| **R-free** | 0.2639 (0.3343) |  |  |  |  |  |  |
| **CC (work)** | 0.952 (0.741) |  |  |  |  |  |  |
| **CC (free)** | 0.928 (0.636) |  |  |  |  |  |  |
| **Number of non-hydrogen atoms** | 9835 |  |  |  |  |  |  |
| **macromolecules** | 9006 |  |  |  |  |  |  |
| **ligands** | 121 |  |  |  |  |  |  |
| **solvent** | 708 |  |  |  |  |  |  |
| **Protein residues** | 1176 |  |  |  |  |  |  |
| **RMS (bonds)** | 0.019 |  |  |  |  |  |  |
| **RMS (angles)** | 0.99 |  |  |  |  |  |  |
| **Ramachandran favored (%)** | 96.33 |  |  |  |  |  |  |
| **Ramachandran allowed (%)** | 3.5 |  |  |  |  |  |  |
| **Ramachandran outliers (%)** | 0.17 |  |  |  |  |  |  |
| **Rotamer outliers (%)** | 2.25 |  |  |  |  |  |  |
| **Clashscore** | 3.62 |  |  |  |  |  |  |
| **Average B-factor** | 32.2 |  |  |  |  |  |  |
| **macromolecules** | 32.44 |  |  |  |  |  |  |
| **ligands** | 27.05 |  |  |  |  |  |  |
| **solvent** | 30.01 |  |  |  |  |  |  |

**Table S2** | Amino acid residue composition of fragment binding sites.

| **S1** | **S2** | **S3** | **S4** | **S5** | **S6** | **S7** | **S8** | **S9** | **S10** | **S11** | **S12** | **S13** | **S14** | **S15** | **S16** |
| --- | --- | --- | --- | --- | --- | --- | --- | --- | --- | --- | --- | --- | --- | --- | --- |
| D137a | E140a | Y296a | V316a | G6a | K32a | K408a | Q167a | K262a | K561a | Y138a | E403a | T147a | K356a | R317b | F285a |
| Y138a | P141a | R322a | S318a | T7a | K33a | V416a | L170a | I292a | D565a | Y223a | G406a | S195a | D359a | L346b | G287a |
| V139a | T142a | G323a | I319a | W10a | D36a | C417a | H173a | G293a | T579a | L224a | V407a | V198a | N362a | C347b | K288a |
| E140a | P143a | F324a | L320a | T47a | V37a | T418a | A174a | A294a | T580a | N225a | K408a | E199a | K364a | V348b | G309a |
| P141a | R260a | G437a | L321a | I48a | E40a | Q422a | D177a | S295a | L581a | A226a | T424a | Q202a |  | H373b | G310a |
| T147a | P261a | K438a | Q326a | L53a | L89a | T423a | Y335a | M315a | H582a | K227a |  | S281a |  | Y374b | D311a |
| N225a | Y263a | P439a | A329a | N55a | Y92a | T424a | P476a | V316a | K561a | I238a |  | L282a |  | T375b | G340a |
| A226a | P264a | Q440a | E330a | I59a |  | V425a | L477a | R317a | D565a | T239a |  | P283a |  |  | K342a |
| K227a | E265a | L441a | G333a | Y129a |  | S426a | E478a | S318a |  | D240a |  |  |  |  | E385a |
| T239a | V270a | V469a | D334a | R454a |  |  | P507a | C347a |  | K241a |  |  |  |  |  |
| D240a | K399a | A470a | E337a | A458a |  |  | E509a | V348a |  | K244a |  |  |  |  |  |
| K241a |  | T471a | F343a | G459a |  |  | W510a | P349a |  | V245a |  |  |  |  |  |
| N242a |  | T472a | K345a | A460a |  |  | R515a | A390a |  | S246a |  |  |  |  |  |
| Q243a |  | V473a | L346a | T461a |  |  | G541a | V391a |  |  |  |  |  |  |  |
| K399a |  |  |  |  |  |  | P542a | G392a |  |  |  |  |  |  |  |
|  |  |  |  |  |  |  | N543a | R393a |  |  |  |  |  |  |  |
|  |  |  |  |  |  |  | C574a |  |  |  |  |  |  |  |  |
|  |  |  |  |  |  |  | K163b |  |  |  |  |  |  |  |  |
|  |  |  |  |  |  |  | Q167b |  |  |  |  |  |  |  |  |
|  |  |  |  |  |  |  | L170b |  |  |  |  |  |  |  |  |
|  |  |  |  |  |  |  | L171b |  |  |  |  |  |  |  |  |
|  |  |  |  |  |  |  | A174b |  |  |  |  |  |  |  |  |
|  |  |  |  |  |  |  | N543b |  |  |  |  |  |  |  |  |
|  |  |  |  |  |  |  | E546b |  |  |  |  |  |  |  |  |

**Table S3** | Number of hydrogen bond acceptors, donors and hydrophobic groups of the pockets with DoGSite druggability score above 0.5.

| **Pocket** | **Druggability score** | **H-acceptor** | **H-donor** | **Hydrophobic interactions** | **Volume (Å^3^)** |
| --- | --- | --- | --- | --- | --- |
| P0 | 0.805067 | 168 | 77 | 101 | 2601.05 |
| P1 | 0.806665 | 148 | 66 | 102 | 2499.9 |
| P2 | 0.807569 | 80 | 23 | 50 | 1339.63 |
| P3 | 0.825454 | 48 | 22 | 34 | 929.48 |
| P4 | 0.74593 | 54 | 19 | 38 | 715.13 |
| P5 | 0.626951 | 32 | 18 | 17 | 465.41 |
| P6 | 0.658511 | 32 | 10 | 20 | 398.68 |
| P7 | 0.650559 | 12 | 14 | 31 | 381.85 |
| P8 | 0.505491 | 28 | 14 | 14 | 364.64 |
| P9 | 0.672195 | 28 | 11 | 25 | 340.93 |
| P12 | 0.611388 | 22 | 12 | 19 | 302.5 |
| P13 | 0.512289 | 20 | 6 | 30 | 286.24 |
| P16 | 0.597747 | 28 | 12 | 12 | 263.68 |
| P17 | 0.557921 | 20 | 8 | 23 | 248.96 |
| P18 | 0.539942 | 10 | 12 | 15 | 246.47 |
| P19 | 0.597388 | 28 | 8 | 4 | 219.7 |
| P21 | 0.578139 | 32 | 9 | 4 | 201.92 |


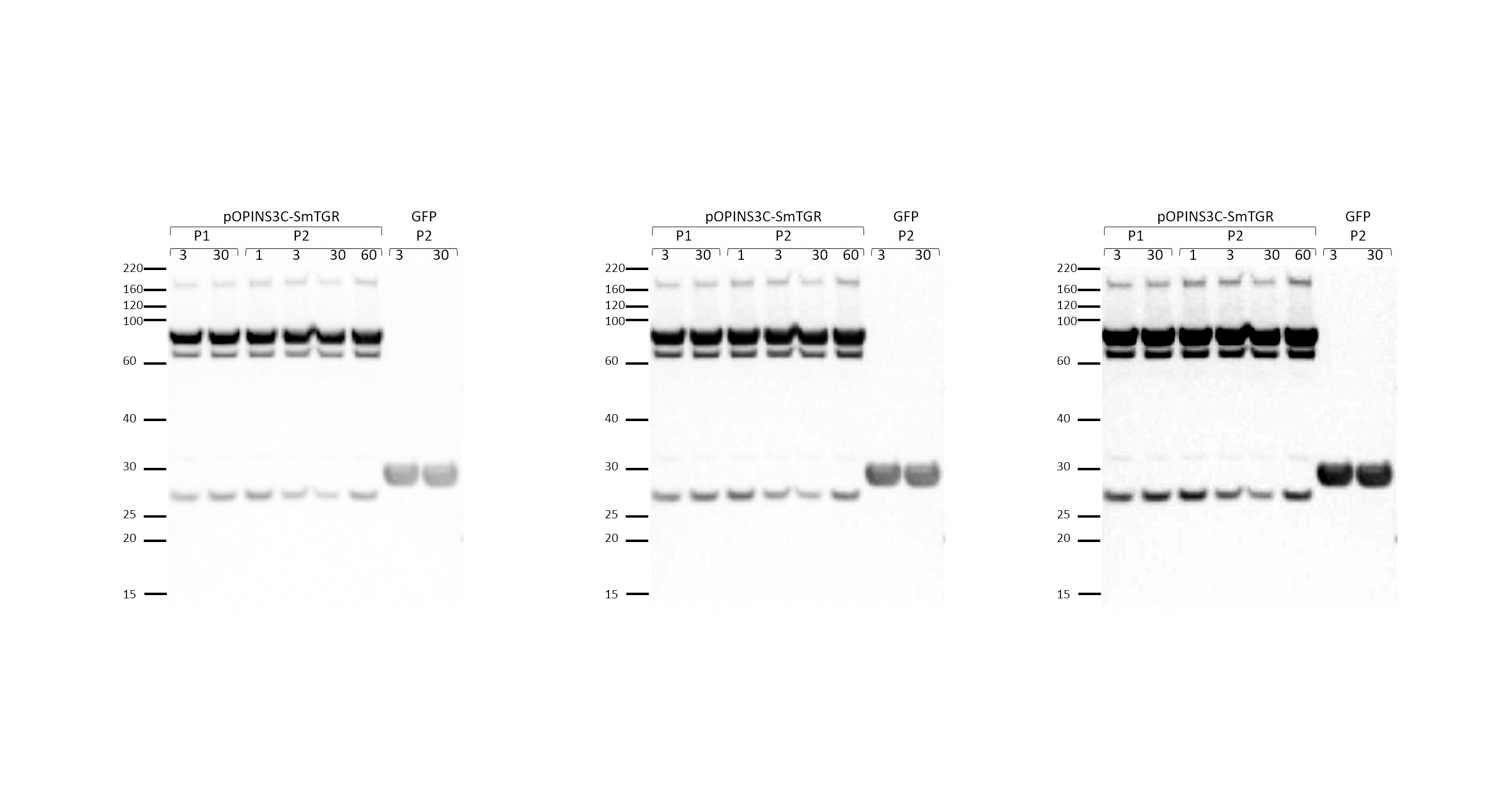


**Figure S1** | Western blot of the intracellular expression of *Sm*TGR in *Sf*9 insect cells analyzed on SDS-PAGE 4-12% and detected with anti-His antibody (Sigma). Multiple exposure images are presented for better visualization. For P1 expression test, 3 and 30 μL from P1 were used to infect 3 mL of 106/mL *Sf*9 cells. For P2 expression test, 1, 3, 30 and 60 μL from P2 were used to infect the same amount of *Sf*9 cells. For control GFP, 3 and 30 μL from P2 were also used to infect *Sf*9 cells as mentioned before.


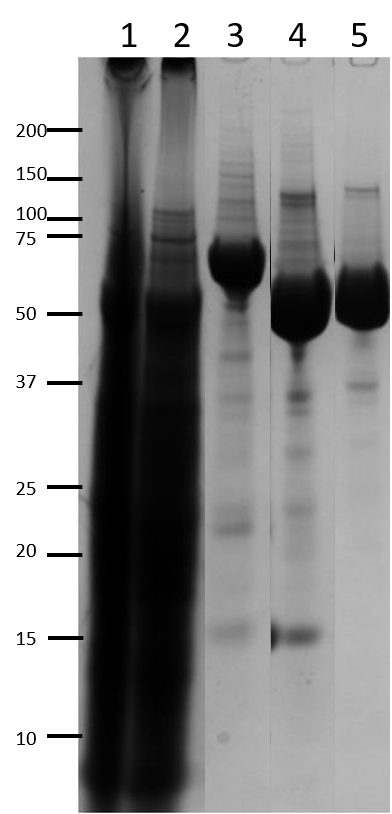


**Figure S2** | SDS-PAGE 4-12 % analysis of *Sm*TGR expressed intracellularly in large-scale culture of *Sf*9 cells, digested and purified. Lane 1, total cell extract load for Ni2+-IMAC; lane 2, flow-through; lane 3, elution; lane 4, flow-through from reverse IMAC of S3C-digested *Sm*TGR; lane 5, SEC fraction of r*Sm*TGR. Note the reduction in size after digestion with S3C protease (lane 4). The estimated purity was 80 %.


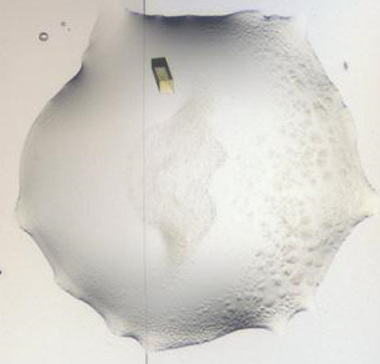


**Figure S3** | *Sm*TGR crystal formed in crystallization solution containing 0.1 M tris pH 8.5, 0.2 M magnesium chloride and 15 % w/v polyethylene glycol 4000. The yellow color is caused by the FAD molecule in its fully reduced form attached to the enzyme.


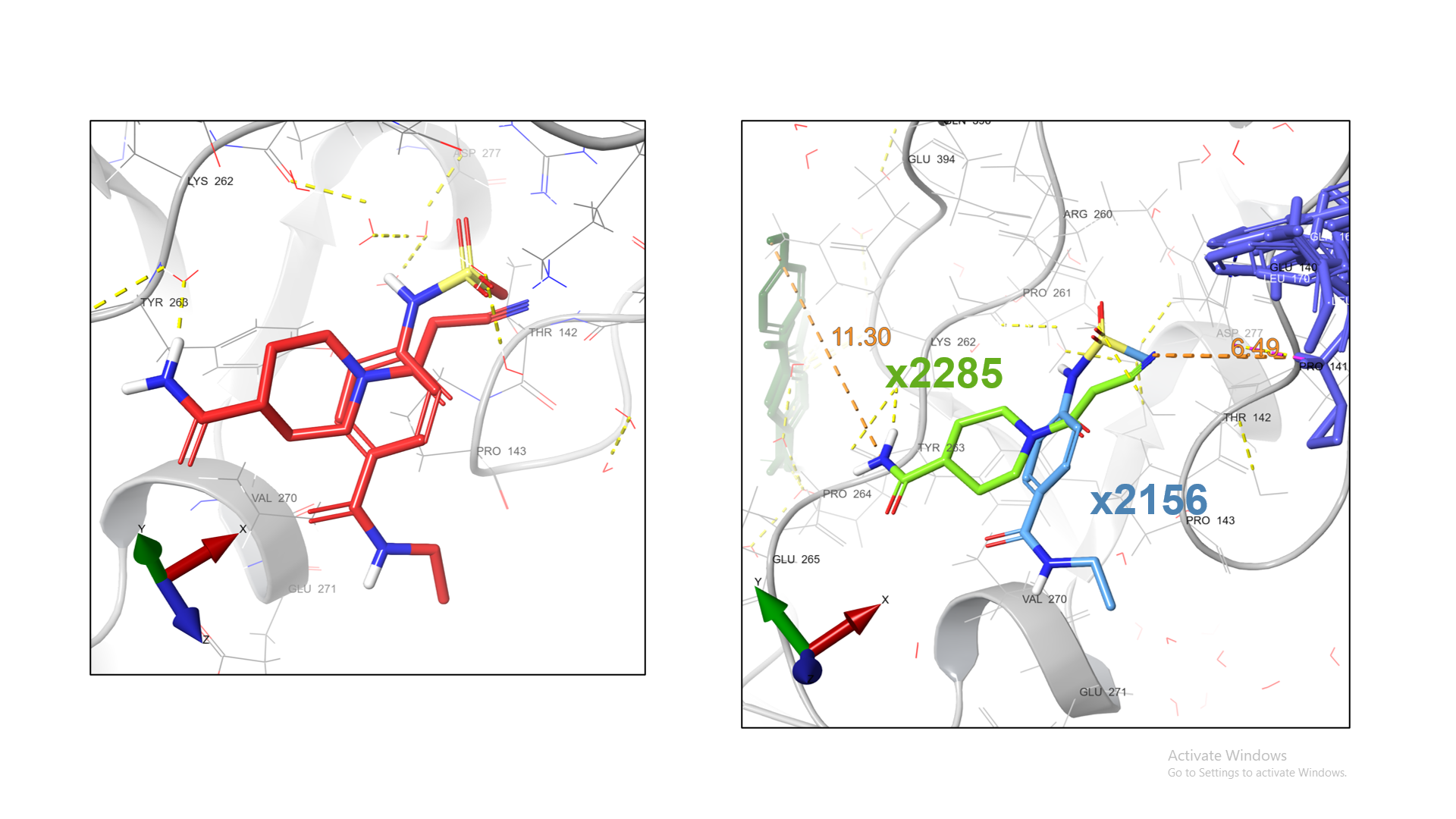


**Figure S4** | Fragments bound to site S2. This binding site was shared by 2 different fragments, the x2285 (light green) and x2156 (light blue). The *Sm*TGR is represented as a gray colored ribbon. The water molecules and protein side chains within a radius of 5 Å are represented by thin sticks in CPK colors with light gray carbons. Hydrogen bonds are represented by dashed yellow lines. The distances from sites 1 (dark blue) and 9 (dark green) are represented as orange dashed lines. Fragment x2285 was seen in a hydrogen bond interaction with residue Tyr263, mediated by a water molecule.


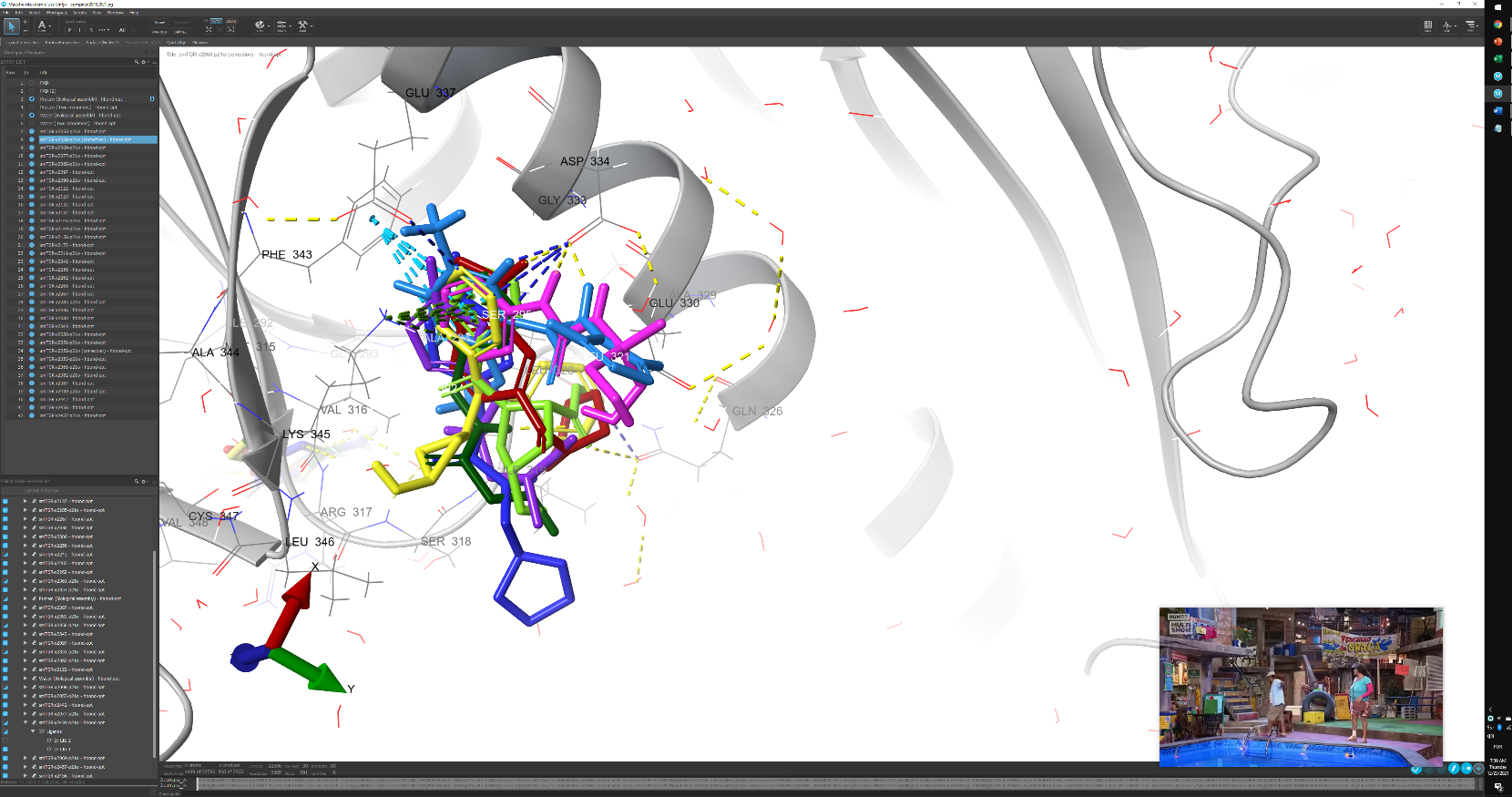

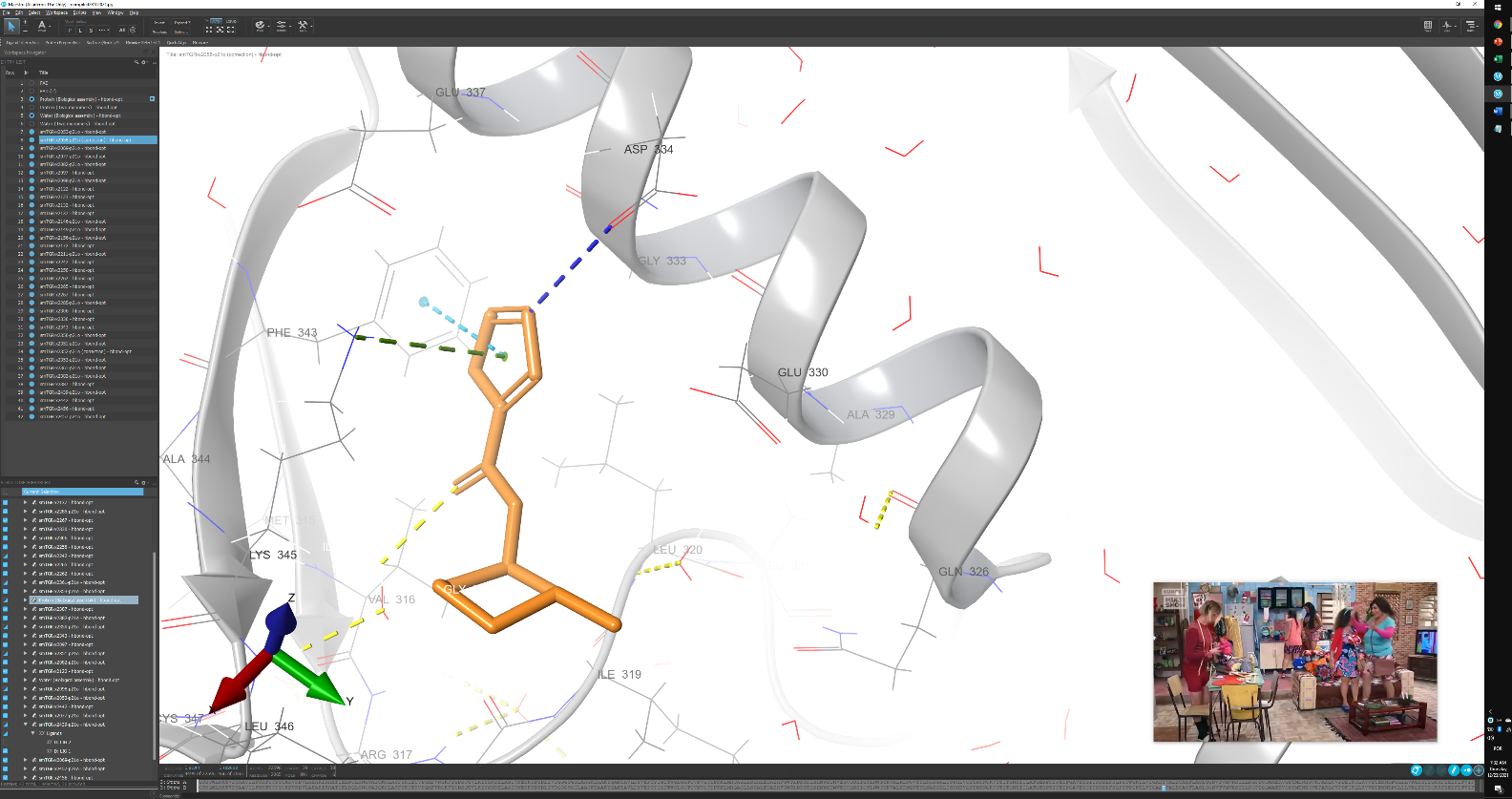


**B**

**A**


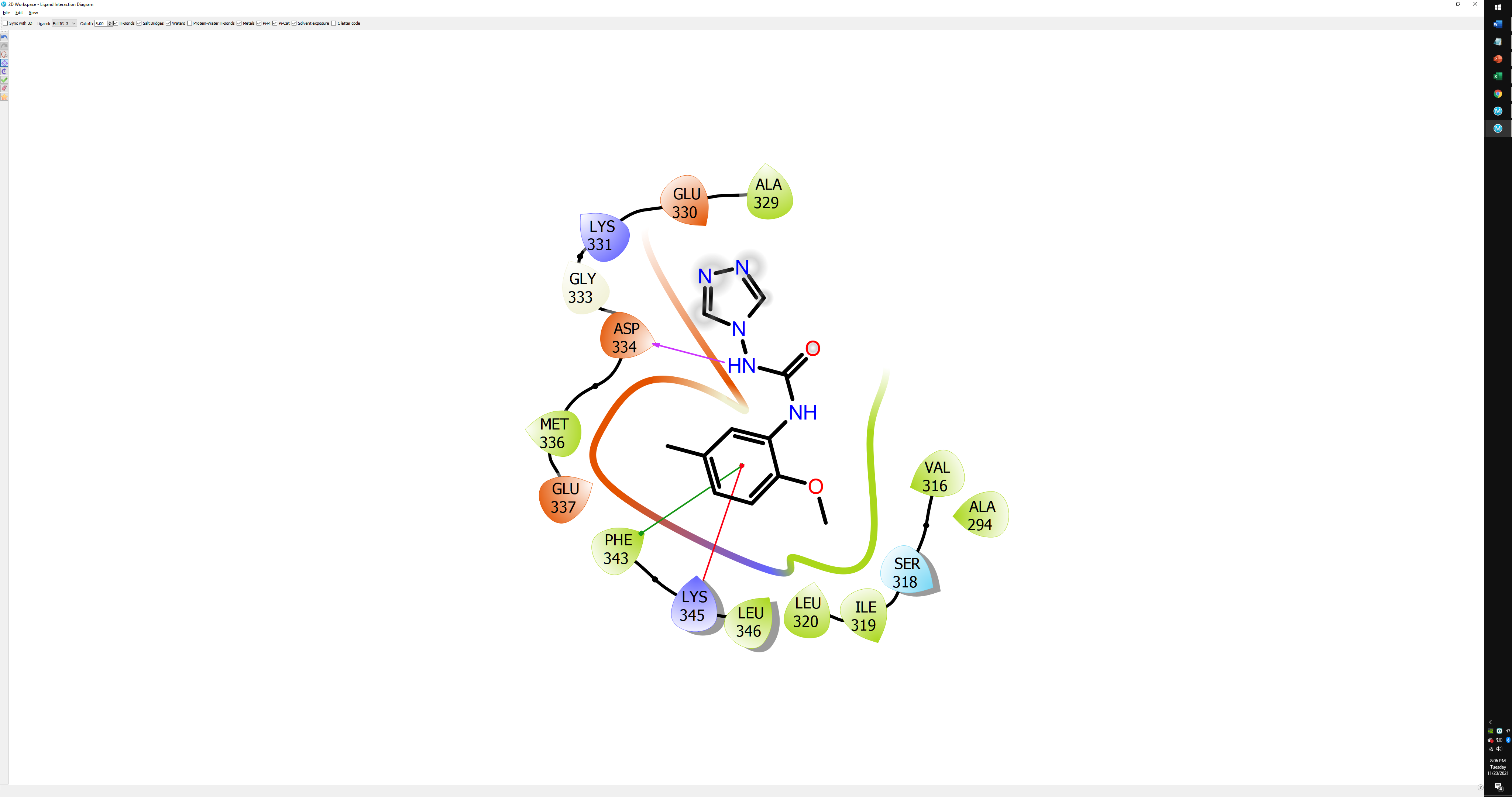


**C**

**Figure S5** | Fragment cluster bound to site S4. These ligands were modelled in *Sm*TGR monomers A (**A**) and B (**B**). Each one of the 9 fragments is represented as thick sticks in different colors. The *Sm*TGR is represented as a gray colored ribbon. The water molecules and protein side chains within a radius of 5 Å are represented by thin sticks in CPK colors with light gray carbons. The hydrogen bonds, π-π and π-cation interactions are represented by yellow, cyan and green dashed lines, respectively. The 2D interaction diagram of fragment x2242 (**C**) depicts the hydrogen bonding, π-π and π-cation (pink, green and red lines, respectively) interactions with Gln243 and Thr239. The pink arrow points towards the hydrogen acceptor atom. We observed 7 π-H interactions between 4 fragments and the enzyme, while hydrogen bonds were observed 3 times in 3 fragments. In this site we also observed a major abundance of π-π and π-cation interactions across the experimental dataset. The π-π stacking and π-cation interactions were observed in 8 and 7 fragments, respectively.


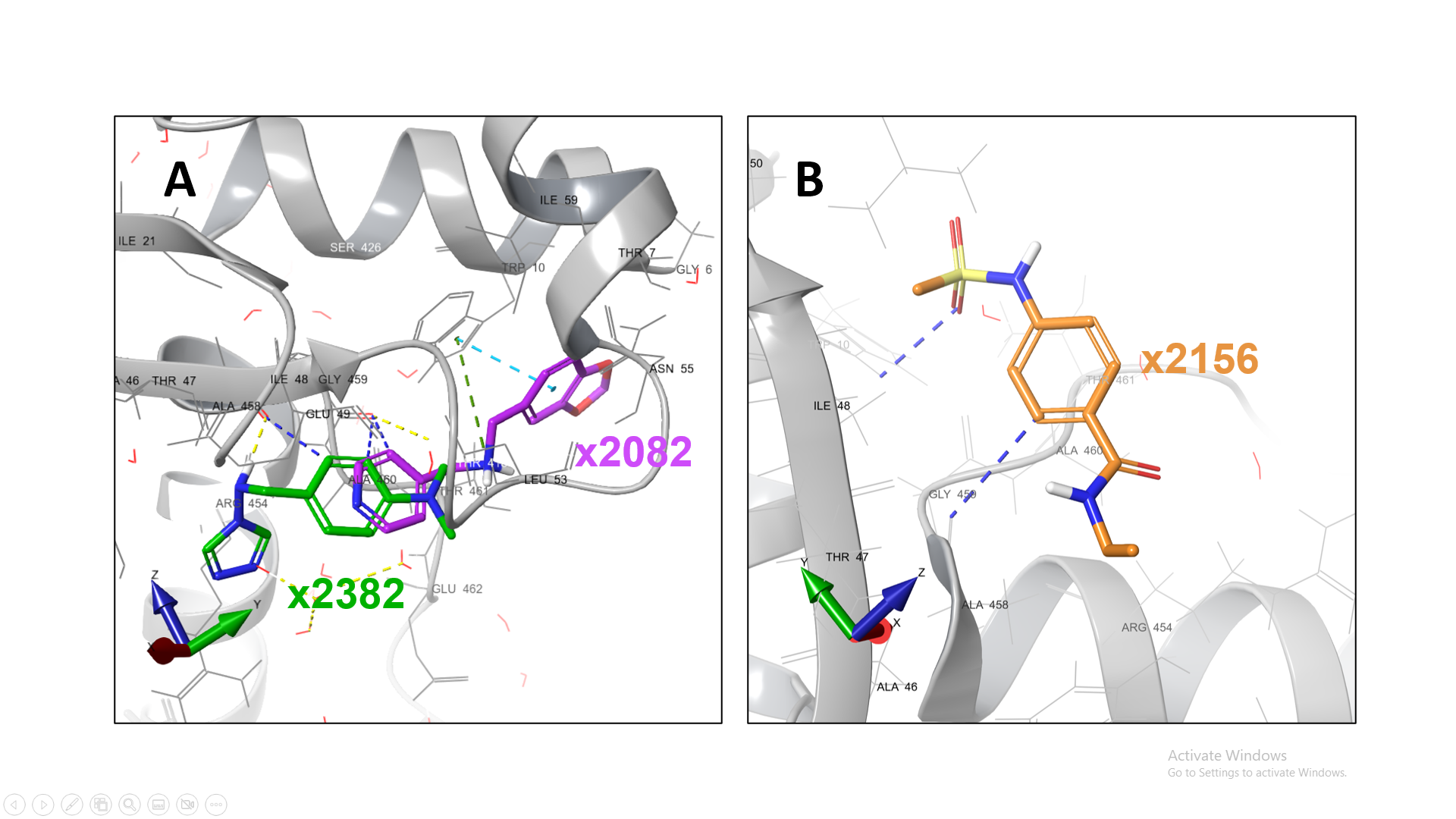


**Figure S6** | Fragments bound to site S5. This binding site is shared by 3 different fragments: x2156 (orange), x2082 (red) and x2382 (green). The fragments forming this cluster were modelled in *Sm*TGR monomers A (**A**) and B (**B**). The *Sm*TGR is represented as a gray colored ribbon. The water molecules and protein side chains within a radius of 5 Å from the fragments are represented by thin sticks in CPK colors with light gray carbons. The hydrogen bonds (yellow), π-H (blue), π-π (cyan) and π-cation (green) interactions are represented by dashed lines in different colors. Fragments x2382 and x2156 showed 2 π-H and x2156 only one. A hydrogen bonding was seen between fragment x2382 and Ala458. Fragment x2082 formed π-π and π-cation with Trp10 residue.


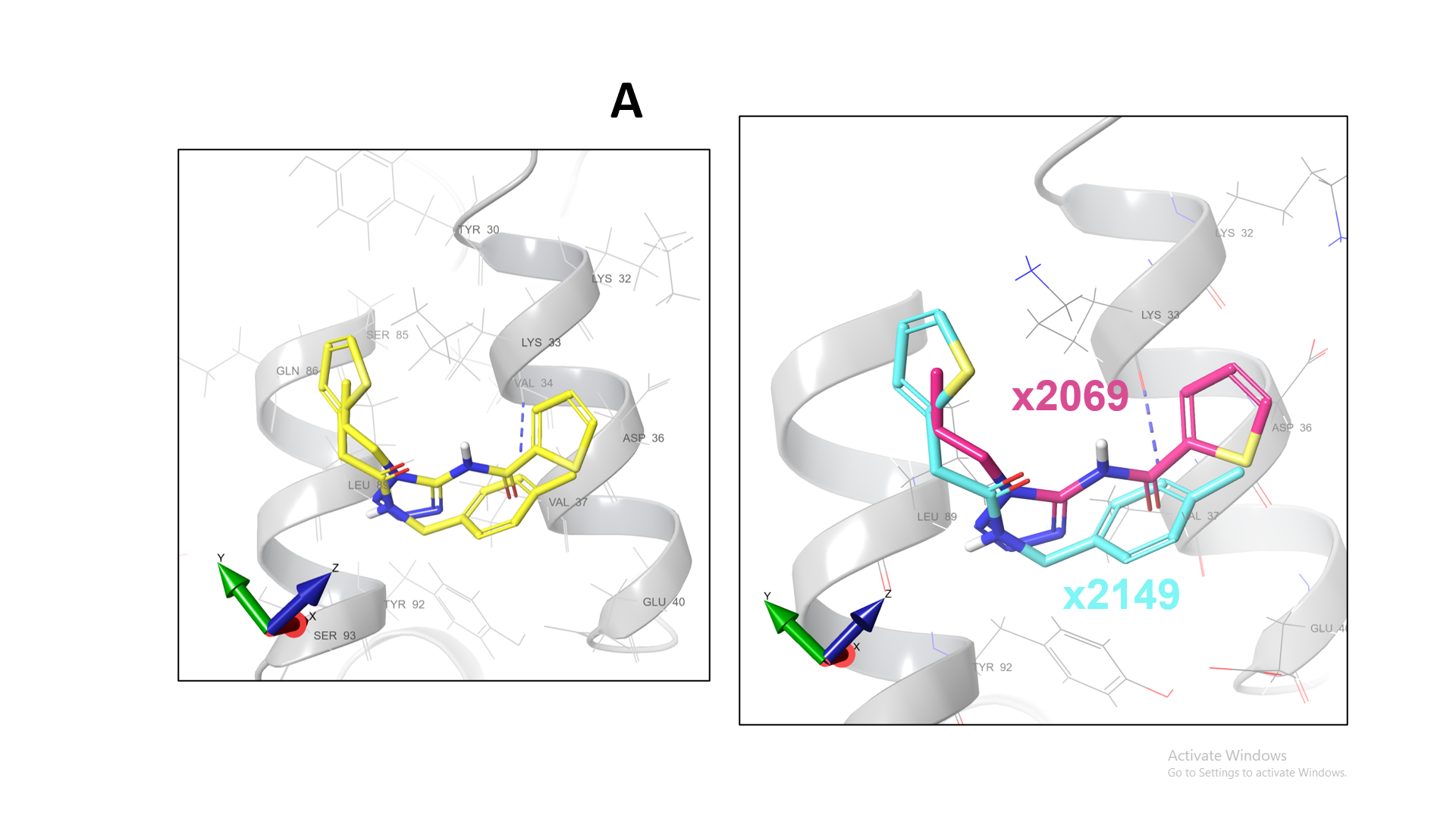


**Figure S7** | Fragments bound to site S6. This binding site was shared between fragments x2149 (cyan) and x2069 (magenta). These fragments are represented as thick sticks in CPK color with different carbon colors. The *Sm*TGR is represented as a gray colored ribbon. The protein side chains within a radius of 5 Å from the fragments are represented by thin sticks in CPK colors with light gray carbons. The π-H is represented by blue dashed lines. Only one π-H interaction was observed between the x2149 fragment and the Lys33.


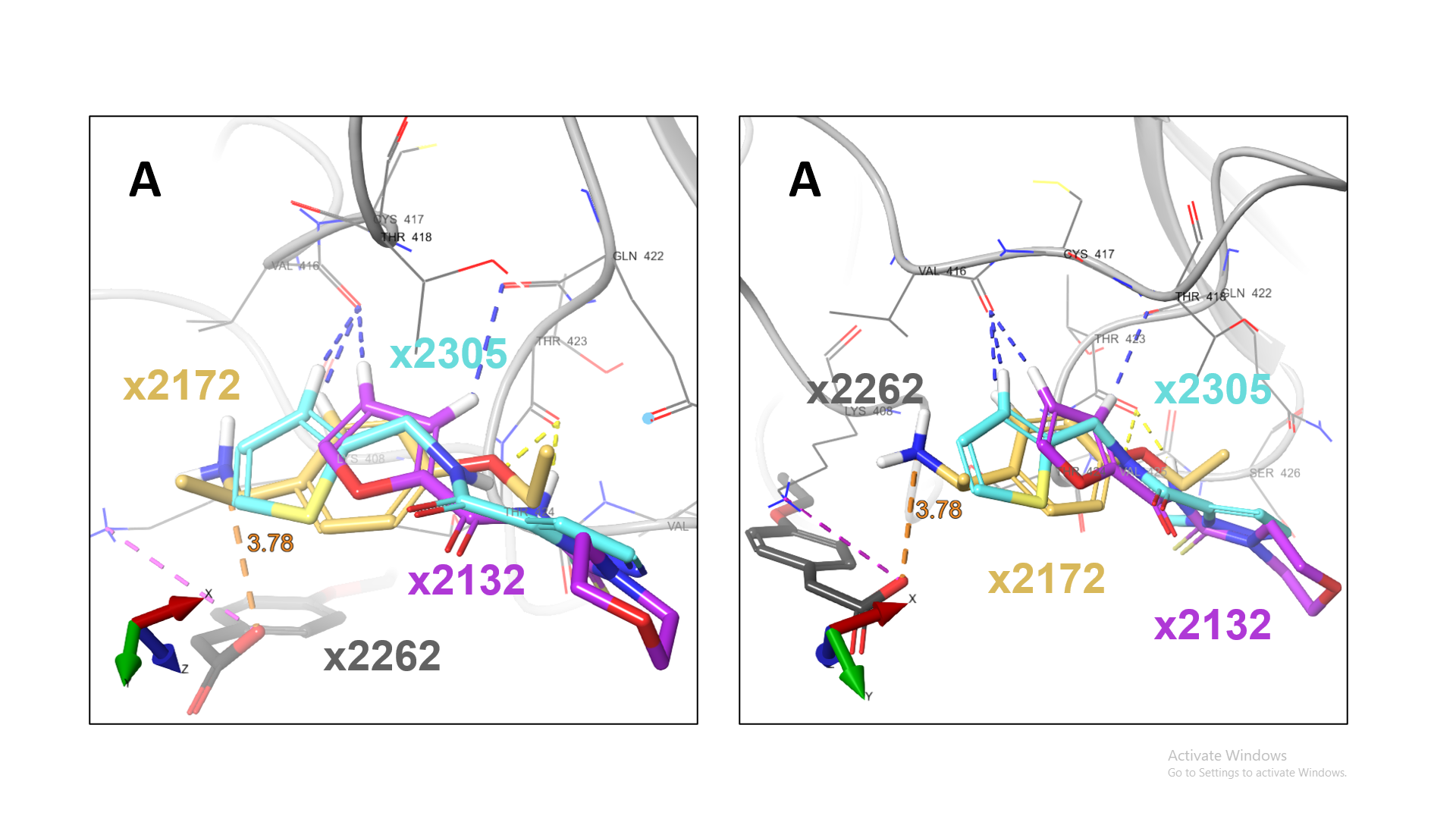


**Figure S8** | Fragments linked to the S7 site. This binding site was common to fragments x2132 (lilac), x2172 (yellow) and x2305 (cyan). These fragments are represented as rods in CPK staining with different carbon colors. *Sm*TGR is represented by a gray ribbon. Protein side chains within a 5 Å radius of the fragments are represented by thin rods in CPK staining, with light gray carbons. The interactions between hydrogen bonds (yellow), π-H (blue) and salt bridge (magenta) are represented by dashed lines. The orange dashed line represents the distance (3.78 Å) between x2172 and x2262 (S12).


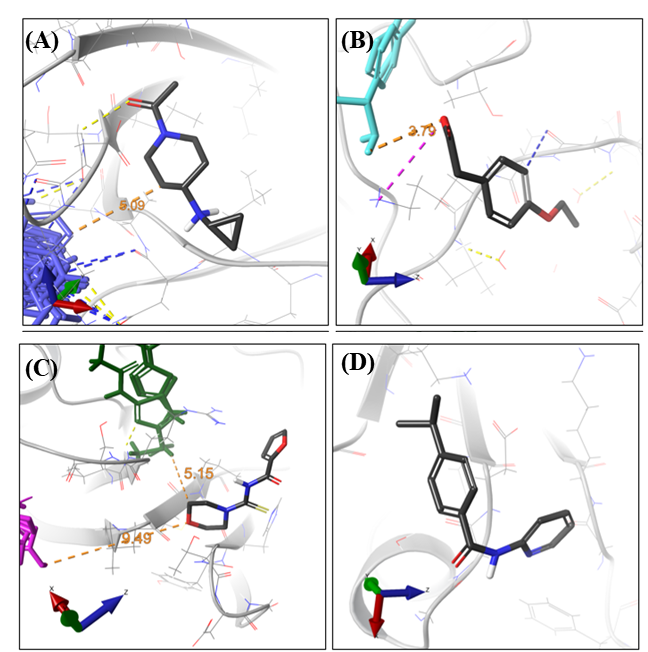


**Figure S9** | Some fragments associated with *Sm*TGR sites binding to a single fragment molecule. (**A**) Fragment x2098 (S10) interactions and its relative location to S1. (**B**) Fragment x2262 (S11) interactions and its relative location to binding S7. (**C**) Fragment x2132 (S14) interactions and relative location to binding S4 (magenta) and S9 (dark green). (**D**) Fragment x2387 (S15). The *Sm*TGR is represented as a gray colored ribbon while the fragment is represented as thick colored sticks in CPK color pattern with dark gray carbons. The side chain of residues within a radius of 5 Å away from the fragments are represented by thin gray sticks. The π-H bonding, H bonding and salt bridges are represented by blue, yellow, and magenta dashed lines, respectively. The distance is represented as an orange dashed line. In S10, the only interaction seen was a hydrogen bond between fragment x2098 carbonyl group and the side chain of the Asp240 residue. In site S12, the fragment x2267 showed two interactions with its binding site. One of these is a π-H interaction between x2267 aromatic ring and Ser281 side chain. Besides, a salt-bridge between the fragment’s C8 atom and Glu199 was also observed. Fragment x2132 (site 14) is at NADPH subdomain, at the TrxR domain. This fragment shares residues with flanking sites S9 (Arg317, Cys347 and Val348) and S4 (Leu346).

**Figure S10** | Screening of selected fragment hits at 100 µM against *Sm*TGR enzymatic activity.
